# A nonhuman primate model with Alzheimer’s disease-like pathology induced by hippocampal overexpression of human tau

**DOI:** 10.1186/s13195-024-01392-0

**Published:** 2024-01-27

**Authors:** Zhouquan Jiang, Jing Wang, Yongpeng Qin, Shanggong Liu, Bin Luo, Fan Bai, Huiyi Wei, Shaojuan Zhang, Junjie Wei, Guoyu Ding, Long Ma, Shu He, Rongjie Chen, Ying Sun, Yi Chen, Lu Wang, Hao Xu, Xiangyu Wang, Gong Chen, Wenliang Lei

**Affiliations:** 1 Guangdong-Hong Kong-Macau Institute of CNS Regeneration, Jinan University, Guangzhou, 510630 Guangdong China; 2 Department of Neurosurgery, the First Affiliated Hospital, Jinan University, Guangzhou, 510630 Guangdong China; 3 Department of Nuclear Medicine and PET/CT-MRI Centre, the First Affiliated Hospital, Jinan University, Guangzhou, 510630 Guangdong China

**Keywords:** Alzheimer’s disease, Human tau, 3R tau, 4R tau, Tau hyperphosphorylation, Nonhuman primate, Neuroinflammation, Blood vessel damage, Cognitive decline

## Abstract

**Background:**

Alzheimer’s disease (AD) is one of the most burdening diseases of the century with no disease-modifying treatment at this time. Nonhuman primates (NHPs) share genetic, anatomical, and physiological similarities with humans, making them ideal model animals for investigating the pathogenesis of AD and potential therapies. However, the use of NHPs in AD research has been hindered by the paucity of AD monkey models due to their long generation time, ethical considerations, and technical challenges in genetically modifying monkeys.

**Methods:**

Here, we developed an AD-like NHP model by overexpressing human tau in the bilateral hippocampi of adult rhesus macaque monkeys. We evaluated the pathological features of these monkeys with immunostaining, Nissl staining, cerebrospinal fluid (CSF) analysis, magnetic resonance imaging (MRI), positron emission tomography (PET), and behavioural tests.

**Results:**

We demonstrated that after hippocampal overexpression of tau protein, these monkeys displayed multiple pathological features of AD, including 3-repeat (3R)/4-repeat (4R) tau accumulation, tau hyperphosphorylation, tau propagation, neuronal loss, hippocampal atrophy, neuroinflammation, Aβ clearance deficits, blood vessel damage, and cognitive decline. More interestingly, the accumulation of both 3R and 4R tau is specific to NHPs but not found in adult rodents.

**Conclusions:**

This work establishes a tau-induced AD-like NHP model with many key pathological and behavioural features of AD. In addition, our model may potentially become one of the AD NHP models adopted by researchers worldwide since it can be generated within 2 ~ 3 months through a single injection of AAVs into the monkey brains. Hence, our model NHPs may facilitate mechanistic studies and therapeutic treatments for AD.

**Supplementary Information:**

The online version contains supplementary material available at 10.1186/s13195-024-01392-0.

## Background

Alzheimer’s disease (AD) is the most prevalent neurodegenerative disorder worldwide with no curative treatment, which is partially due to the dismal track record of bench-to-bedside translation of intervention strategies developed and evaluated in AD rodent models [1, 2]. Although rodent AD models are powerful tools, their inherent limitations, such as the absence of 3-repeat (3R) tau in adult mice, prevent them from fully reproducing the complete spectrum of brain pathologies associated with AD or related tauopathies [3, 4]. Nonhuman primates (NHPs) have close phylogenetic relationships as well as many physiological and pathological parallels with humans. They have emerged as indispensable translational tools in the identification and validation of early diagnostic markers, as well as in the development and evaluation of safe and effective treatments for AD [5, 6]. To our knowledge, all the NHP models of AD available to date can be categorized into either spontaneous or artificially induced models, and the latter can be further classified as genetically or nongenetically modified models [7]. Many NHPs, including rhesus macaques, mouse lemurs, and common marmosets, exhibit Aβ deposits, incipient tau pathology, and cognitive deficits as they age. Unfortunately, these naturally aged monkeys are not very consistent phenocopies of AD patients, since the process of ageing is essentially different from the progression of AD. Moreover, the limited availability, prohibitive breeding costs, and very long generation period of NHPs significantly restrict the broader application of spontaneous NHP models [8–11]. In accordance with a series of hypotheses of AD onset, NHP models induced by cholinergic neuron injury, Aβ injection, formaldehyde exposure, and streptozotocin injection each reflect certain pathological aspects or behavioural deficits of AD. However, there are continuing controversies regarding the validity of the theoretical foundations of these models. Moreover, the uniformity of model development, side effects of drug administration, penetration damage caused by surgical procedures, and still substantial time frame considerably limit the potential utilization of nongenetically engineered NHP models [12–16]. Today, several genetic NHP models of AD, such as marmosets with edited *PSEN1* and *APP* transgenic cynomolgus monkeys, are looming on the horizon. However, again, these promising models have limitations. Ethical considerations, an underrepresentation of sporadic AD, nontissue-specific gene expression, unpredictable genomic integration, and suboptimal gene transfer efficiency lead to the paucity and undermine the reliability of genetically modified NHP models [17, 18].

To remove the roadblocks preventing the wide application of NHP models in AD research, we attempted to establish a convenient and reliable monkey model to facilitate preclinical studies on AD. Growingly recognized as a major driver of AD, tau captured our attention since it surpasses Aβ in predicting the location of brain atrophy in AD patients and correlates closely with AD progression and cognitive decline [19–22]. In fact, rhesus monkeys that received injections of pathological tau-expressing adeno-associated virus (AAV) in the unilateral entorhinal cortex displayed misfolded tau propagation accompanied by extensive microglial responses and robust alterations in cerebrospinal fluid (CSF) and plasma biomarkers for AD [23]. Moreover, tauopathy can promote spinal cord-dependent production of toxic Aβ in transgenic monkeys [24]. Therefore, in this study, we created a new AD-like NHP model by overexpressing human tau (hTau) in the bilateral hippocampi of middle-aged rhesus monkeys. In these monkeys, AD-like characteristics can be generated within a few months following a single stereotaxic injection of AAVs, yet the monkeys exhibit many defining pathological features of AD, such as the accumulation of 3-repeat (3R)/4-repeat (4R) tau isoforms, the hyperphosphorylation of exogenous and endogenous tau, neuronal loss, inflammatory responses, medial temporal lobe atrophy, elevated Aβ burden, neurofibrillary tangle (NFT) formation, vascular abnormalities, and multidomain cognitive dysfunctions. Many of these AD-like pathologies and cognitive declines were first reported in tau-based NHP models for AD, and the accumulation of both 3R and 4R tau isoforms is an NHP-specific pathological feature that cannot be reproduced in rodent models for AD. Once adopted by the research community, they are expected to add to the arsenal of AD NHP models and to contribute to the fight against this devastating disease.

## Methods

### Reagents and antibodies

The anaesthetics, analgesics, and sedatives we used in this study included Zoletil™ 50 (Virbac, France), Dexdomitor® (dexmedetomidine, Orion Pharma, Finland), propofol (Guangdong Jiabo Pharmaceutical, China), atropine (Shanghai Quanyu Biotechnology, China), and lidocaine (Shandong Hualu Pharmaceutical, China). The primary antibodies we used in this study were as follows: chicken anti-GFP (Abcam, ab13970, 1:1000), rabbit anti-Tau (Dako, A0024, 1:1000), mouse anti-pTau (S202/T205, Invitrogen, MN1020B, 1:500), rabbit anti-pTau (T231, Abcam, 1008030–3, 1:1000), mouse anti-4R Tau (Millipore, 3887010, 1:1000), mouse anti-3R Tau (Millipore, 3916679, 1:1000), guinea pig anti-NeuN (Millipore, ABN90, 1:1000), mouse anti-Tuj1 (BioLegend, 801202, 1:1000), rabbit anti-MAP2 (Millipore, AB5622, 1:500), rat anti-GFAP (Invitrogen, 13–0300, 1:1000), rabbit anti-Iba1 (Fujifilm, 019–19741, 1:1000), rabbit anti-CD68 (Abcam, ab125212, 1:500), mouse anti-Aβ (BioLegend, 800701, 1:500), rabbit anti-laminin (Sigma, L9393, 1:1000), rabbit anti-AQP4 (Proteintech, 16473–1-AP, 1:500), and mouse anti-PECAM-1 (Thermo Fisher, MA5-13188, 1:1000). The secondary antibodies we used in this study were as follows: donkey anti-mouse, rat, rabbit, chicken, and guinea pig conjugated to Alexa Fluor 488, 555, and 647 (Invitrogen or Jackson ImmunoResearch, A11039, A21206, A21208, A31570, A31572, A21435, A21207, A31571, A21247, 712–165-150, 711–605-152, 706–605-148, 1:1000 or 1:500). DAPI and thioflavin-S were obtained from Roche (10236276001, 1:1000) and Sigma (T1892-25G, 1:5000), respectively.

### AAV preparation

The hTau (4R isoform) gene was packaged into the recombinant AAV serotype 9 (rAAV9) used for monkey hippocampal injection in this study. The control AAV refers to rAAV9 CAG::FRT-hTau, which does not express hTau without FLPo/FRT-mediated recombination. The hTau-expressing AAVs refer to rAAV9 Syn::FLPo and rAAV9 CAG::FRT-hTau, which overexpress site-specific recombinase FLPo under the neuron-specific synapsin promoter and then induce hTau expression under the ubiquitous CAG promoter. All AAVs used in this study were produced by PackGene Biotech Inc. (Guangzhou, China). Briefly, the AAV production workflow consisted of several stages, including bacterial transformation and preparation, plasmid preparation, HEK 293 T cell cotransfection (GOI, pRep2CapX, and pHelper plasmids), crude purification, concentration and ultracentrifugation (iDGU), sterilization (0.2 μm filter), and quality control tests. The AAV quality control assessments included restriction digestion analysis with endonucleases to verify the AAV packaging plasmids; AAV titering by quantitative PCR (qPCR; SYBR Green with standard curve); endotoxin testing by limulus amoebocyte lysate (LAL) assay; and AAV purity analysis by SDS‒PAGE and Coomassie blue and silver staining.

### Animals

Eighteen adult rhesus macaques (*Macaca mulatta*, sixteen males and two females, 7 to 15 years old, body weights: 4 ~ 8 kg, Table 1) were used in this study. The macaques were maintained in accordance with the standards set forth in the 8th edition of the *Guide for the Care and Use of Laboratory Animals* (NRC, 2011). All of them were housed in cages that can be easily sanitized and placed in climate-controlled rooms at Guangdong Yuan Xi Biotech Co., Ltd. (Guangzhou, China), which was fully accredited by the Association for Assessment and Accreditation of Laboratory Animal Care (AAALAC) International, under the close supervision of three laboratory animal technicians and the veterinarians of the institute. All experimental procedures were approved by the Institutional Animal Care and Use Committee (IACUC) of Guangdong Yuan Xi Biotech Co., Ltd. (IACUC No. YXSW-2021–002) and Jinan University (IACUC No. 20210806–03) and were in full compliance with the “Guide for the Care and Use of Laboratory Animals of the Institute of Laboratory Animal Science (est. 2006)” and “The use of nonhuman primates in research of the Institute of Laboratory Animal Science (est. 2006)” to ensure the safety of personnel and animal welfare.

**Table 1:** Animal information summary

Animal No	Sex	Age (year)	Virus injection	The experiments that the animal was used for
07379	M	16	AAV expressing hTau + AAV expressing GFP	Immunostaining (GFP), Immunostaining (hTau/pTau/3R Tau/4R Tau), CSF collection & analysis (AD biomarkers), PET imaging (Tau/FDG), MRI (hippocampal atrophy), Thioflavin S staining, Behavioral tests (learning, memory)
08019	M	15	AAV expressing hTau + AAV expressing GFP	Immunostaining (GFP), Immunostaining (hTau/pTau/3R Tau/4R Tau), CSF collection & analysis (AD biomarkers), PET imaging (Tau/FDG), MRI (hippocampal atrophy), Thioflavin S staining, Behavioral tests (learning, memory)
08061	M	15	AAV expressing hTau	Immunostaining (hTau/pTau/3R Tau/4R Tau), PET imaging (Tau/FDG), Immunostaining (NeuN/Tuj1/MAP2/GFAP/Iba1/CD68/Laminin/PECAM1/AQP4), Nissl staining, Thioflavin S staining
08383	M	15	AAV expressing hTau + AAV expressing GFP	Immunostaining (hTau/pTau/3R Tau/4R Tau), CSF collection & analysis (AD biomarkers), PET imaging (Tau/FDG), MRI (hippocampal atrophy), Thioflavin S staining, Behavioral tests (learning, memory)
09016	F	14	AAV expressing hTau + AAV expressing GFP	Immunostaining (GFP), Immunostaining (hTau/pTau/3R Tau/4R Tau), CSF collection & analysis (AD biomarkers), PET imaging (Tau/FDG), MRI (hippocampal atrophy), Thioflavin S staining, Behavioral tests (learning, memory)
09078	F	14	AAV expressing hTau + AAV expressing GFP	Immunostaining (GFP), Immunostaining (hTau/pTau/3R Tau/4R Tau), CSF collection & analysis (AD biomarkers), PET imaging (Tau/FDG), MRI (hippocampal atrophy), Thioflavin S staining, Behavioral tests (learning, memory)
14189	M	9	AAV expressing hTau + AAV expressing GFP	Immunostaining (GFP), Immunostaining (hTau/pTau/3R Tau/4R Tau), PET imaging (Tau/FDG), Immunostaining (NeuN/Tuj1/MAP2/GFAP/Iba1/CD68/Laminin/PECAM1/ AQP4), Nissl staining
16167	M	7	AAV expressing hTau + AAV expressing GFP	Immunostaining (GFP), Immunostaining (hTau/pTau/3R Tau/4R Tau), PET imaging (Tau/FDG), Immunostaining (NeuN/Tuj1/MAP2/GFAP/Iba1/CD68/Laminin/PECAM1/ AQP4)
16195	M	7	Control AAV + AAV expressing GFP	Immunostaining (GFP), Immunostaining (hTau/pTau/3R Tau/4R Tau), Immunostaining (NeuN/Tuj1/MAP2/GFAP/Iba1/CD68/Laminin/PECAM1/AQP4), Nissl staining
16197	M	7	Control AAV + AAV expressing GFP	Immunostaining (GFP), Immunostaining (hTau/pTau/3R Tau/4R Tau), Immunostaining (NeuN/Tuj1/MAP2/GFAP/Iba1/CD68/Laminin/PECAM1/AQP4), Nissl staining
134021	M	10	AAV expressing hTau	Immunostaining (hTau/pTau/3R Tau/4R Tau), Immunostaining (NeuN/Tuj1/MAP2/ GFAP/Iba1/CD68/Laminin/PECAM1/AQP4), Nissl staining
134023	M	10	AAV expressing hTau	Immunostaining (hTau/pTau/3R Tau/4R Tau), Immunostaining (NeuN/Tuj1/MAP2/ GFAP/Iba1/CD68/Laminin/PECAM1/AQP4), Nissl staining
134041	M	10	AAV expressing hTau	Immunostaining (hTau/pTau/3R Tau/4R Tau), Immunostaining (NeuN/Tuj1/MAP2/ GFAP/Iba1/CD68/Laminin/PECAM1/AQP4), Nissl staining
08111401	M	15	Control AAV	Immunostaining (hTau/pTau/3R Tau/4R Tau), Thioflavin S staining, Behavioral tests (learning, memory)
09185	M	14	Control AAV	Immunostaining (hTau/pTau/3R Tau/4R Tau), Thioflavin S staining, Behavioral tests (learning, memory)
13041923	M	10	Control AAV	Immunostaining (hTau/pTau/3R Tau/4R Tau), Immunostaining (NeuN/Tuj1/MAP2/ GFAP/Iba1/CD68/Laminin/PECAM1/AQP4), Nissl staining, Thioflavin S staining, Behavioral tests (learning, memory)
14102103	M	9	Control AAV	Immunostaining (hTau/pTau/3R Tau/4R Tau), Immunostaining (NeuN/Tuj1/MAP2/ GFAP/Iba1/CD68/Laminin/PECAM1/AQP4), Nissl staining, Thioflavin S staining, Behavioral tests (learning, memory)
16030703	M	7	Control AAV	Immunostaining (hTau/pTau/3R Tau/4R Tau), Immunostaining (NeuN/Tuj1/MAP2/ GFAP/Iba1/CD68/Laminin/PECAM1/AQP4), Nissl staining, Thioflavin S staining, Behavioral tests (learning, memory)

This table lists the monkey information, including animal no., sex, age, the viruses that were injected into the hippocampus, and the experiments that each monkey was used for. A total of 18 adult *Macaca mulattas* (16 males and 2 females, 7 to 15 years old, body weights: 4 ~ 8 kg) were used in this study. Control AAV: AAV CAG::FRT-hTau; AAV expressing hTau: AAV Syn::FLPo + AAV CAG::FRT-hTau; AAV expressing GFP: AAV Syn::GFP

### Surgical procedures and stereotactic injection of AAVs

All monkeys were fasted for ~ 12 h prior to general anaesthesia to help prevent vomiting and the aspiration of stomach contents while they were under general anaesthesia. Atropine (0.02 mg/kg, intramuscular injection) was given preoperatively to reduce the secretions in the mouth and respiratory passages of the monkeys. Anaesthesia was induced by Zoletil™ 50 (tiletamine and zolazepam, 5–10 mg/kg, intramuscular) and Dexdomitor® (dexmedetomidine, 10–20 mcg/kg, intramuscular). During surgery, anaesthesia was maintained with propofol (2.5–3.5 mg/kg, intravenous), while lidocaine (1–4 mg/kg, subcutaneous, intramuscular) was also administered as a local anaesthetic before the operation to further reduce pain.

For hippocampal AAV injection, the monkeys were placed on a heated V-top surgical table with their heads stabilized on a stereotaxic frame (RWD Life Science, Shenzhen, China). The precise positions (6 within each hippocampus) for stereotaxic injection were determined based on the T1-weighted MRI scans of the monkey brains. A hand-held cranial microdrill (RWD Life Science, Shenzhen, China) was used to drill several small holes in the skull to allow the injection needles to pass through. Then, 7.5 µl of AAVs (10^11~12^ GC/ml, flow rate: 800 nl/min) were injected into each injection site using a 25-µl microsyringe (Hamilton Company, USA) controlled by a Micro4™ controller and an UltraMicroPump 3 (World Precision Instruments, USA). After each injection, the microsyringe needle was kept in place for 10 additional minutes before it was slowly withdrawn. During the surgical procedures, the blood oxygen level (> 95%), heart rate (150–220/min), respiratory rate (10–25/min), and blood pressure (> 60 mmHg mean value and > 90 mmHg for systolic pressure) were all monitored and the data were collected with a paediatric vital signs monitor (JRTYL, Hunan, China). Finally, the muscles and skin around the wound were repeatedly cleaned and sutured, and after the operation, penicillin sodium (100,000 U/kg, intramuscular) was given for three consecutive days to prevent wound infection.

### Immunohistochemistry (IHC)

The monkeys were euthanized via a sodium pentobarbital overdose (100 mg/kg) and transcardially perfused first with ice-cold PBS and later with a mixture of paraformaldehyde (PFA; 4%) and sucrose (10%). After perfusion, whole monkey brains were dissected, sliced into 1-cm coronal blocks with a monkey brain matrix (Shanghai Tow Intelligent Technology, China), and then postfixed and dehydrated in 4% PFA (48 h) and gradient sucrose solutions (10, 20 and 30%). The dehydrated brain blocks were embedded in Tissue-Tek® O.C.T. Compound (Sakura Finetek, USA) and then serially sectioned in the coronal plane on a cryostat (CryoStar™ NX50, Thermo Fisher Scientific, USA) at 50-mm thickness.

For immunofluorescence, free floating brain sections were first washed with PBS and blocked in blocking solution (5% normal donkey serum, 3% BSA, and 0.5% Triton X-100 in PBS) for 1 h at room temperature and then incubated overnight at 4 °C with primary antibodies diluted in blocking solution. After at least 3 washes with PBS, the brain sections were incubated with DAPI and appropriate secondary antibodies conjugated to Alexa Fluor 488, Alexa Fluor 555, or Alexa Fluor 647 for 2 h at room temperature, followed by extensive washing with PBS. Finally, the brain sections were mounted with VECTASHIELD® antifade mounting medium (VECTOR Laboratories, USA) and sealed with nail polish.

### Wide-field and confocal fluorescence imaging and the quantification of IHC results

Most of the fluorescence images were acquired with modular microscope user interface software (64 bit version of ZEN 2.5, Carl Zeiss, Jena, Germany) on either a conventional wide-field epifluorescence microscope (Carl Zeiss Axio Imager Z2, Jena, Germany) or a confocal laser scanning microscope (Carl Zeiss LSM 880 with Airyscan, Jena, Germany).

For the brain sections being directly compared, all the IHC processes were performed simultaneously, and all the image acquisition settings on the microscopes were identical. To quantify the cells bearing certain markers, the 2-dimensional (2D) cell density (cells per mm^2^) was calculated by dividing the cell number in a region of interest (ROI) by the total area of the ROI, and the 3-dimensional (3D) cell density (cells per mm^3^) was calculated by multiplying the 2D cell density by the number of brain slices needed to make a stack 1 mm thick. To ensure a systematic random sampling procedure for a certain IHC marker, we chose 9 (3 × 3) or 16 (4 × 4) symmetrically and evenly distributed ROIs (with each ROI containing at least 100 + certain cells) in a certain brain region of a brain slice, and we chose 5 ~ 10 evenly spaced (at 400 ~ 2000 μm, depending on the size of the brain region) brain slices from each animal. Last, each N in this study represents the number of independent animals.

### MRI and PET scans

All MRI scans were performed on a 3.0-T MRI scanner (Discovery MR750 3.0 T, GE Healthcare, USA) equipped with an 8-channel customized head coil for macaques (Medcoil MK80, Suzhou, China) at the PET/CT-MRI centre in the First Affiliated Hospital of Jinan University. Prior to each MRI scan, the monkeys were fasted for at least 6 h and anaesthetized with an intramuscular injection of Zoletil™ 50 and Dexdomitor®. When the MRI data were collected to determine the coordinates for stereotaxic injection of AAVs, a pair of glass capillary tubes (World Precision Instruments, USA) filled with glycerol was fixed onto the top of the skull ahead of the scans to serve as spatial reference points. The whole-brain images were acquired with a 3D Bravo T1 sequence (TR = 8.4 ms, TE = 3.5 ms, slice thickness = 0.5 mm, matrix size = 300 × 300, FOV = 15 × 15 cm), a CUBE T2 sequence (TR = 2500.0 ms, TE = 108.9 ms, slice thickness = 0.5, matrix size = 320 × 320, FOV = 15 × 15 cm), and a T2 FLAIR sequence (TR = 8400.0 ms, TE = 151.7 ms, slice thickness = 1.0 mm, matrix size = 256 × 256, FOV = 16 × 16 cm).

All ^18^F-FDG and ^18^F-T807 PET scans were conducted using a 128-slice time-of-flight PET/CT scanner (GE Discovery PET/CT 690 Elite, GE Healthcare, USA) at the PET/CT-MRI centre in the First Affiliated Hospital of Jinan University. Before each PET scan, the macaques were fasted and anaesthetized as previously mentioned. The radiochemical purities of both ^18^F-FDG and ^18^F-T807 were over 99%. Each monkey was intravenously injected with ^18^F-FDG (approximately 150 MBq, 0.3 ~ 0.5 mCi/kg) or ^18^F-T807 (approximately 150 MBq, 0.3 ~ 0.5 mCi/kg) while awake via the posterior saphenous vein. After 50 min, the macaque was placed into the scanner. Head position was fixed with a stereotactic frame, and a static PET scan was performed. Protocols: A CT scan was performed first, followed by a 10-min static positron emission data collection at 60 min postinjection. The PET data were attenuation-corrected by integrated CTAC technology. The PET/CT and MR images were coregistered and analysed with PMOD software. The standard uptake value (SUV) of each ROI, which was decay corrected back to the radioligand injection time point, was quantitatively extracted based on an individual atlas.

### Animal behavioural studies

The delayed response (DR) task was conducted using the Wisconsin General Test Apparatus (WGTA), in which a monkey sat in its home cage in front of a tray that contained 3 food wells covered by identical swing-away lids. Initially, the experimenter baited one of the wells with food in front of the monkey, covered it, and then lowered an opaque screen to block the food tray from the monkey’s view. After a certain delay period (“memory retention interval”), the screen was removed, which allowed the monkey to retrieve the food from the baited food well, which had to be recalled from working memory. Food reinforcers were randomly distributed among 3 wells over 30 trials per day. For each monkey, the initial memory retention interval was set to 5 s, and the memory retention interval was gradually increased according to a stepwise procedure every time the monkey made ≥ 26 correct choices out of 30 trials per day for 3 consecutive days (“master a memory retention interval”). When a monkey was not able to master a memory retention interval in a week, the DR task was terminated, and the previous memory retention interval the monkey had mastered was recorded for subsequent analysis. The task was performed with the same protocol for all monkeys in the control and hTau-overexpressing groups pre- and postoperatively, and most monkeys took ~ 4 weeks to be able to complete the task.

The delayed matching-to-sample (DMTS) task was also performed with a WGTA. The experimenter first presented a sample visual stimulus (a katakana character or LEGO® block) to the monkey. Following a random interval (up to 5 s), the same sample visual stimulus and another visual distractor were both presented to the monkey, and the monkey needed to choose the visual stimulus that matched the sample stimulus to obtain the food reward. Each incorrect choice was followed by a 10-s timeout. The monkeys underwent 30 DMTS trials per day, and both the sample visual stimuli and the visual distractors were randomly selected each day from either 46 basic katakana alphabet letters or a large pool of LEGO® blocks (> 200 combinations). The monkeys were considered to have mastered the task when they made ≥ 26 correct choices out of 30 trials per day for 3 consecutive days. The time a monkey required to master the task (“learning period”) was recorded for subsequent analysis. The delayed nonmatching-to-sample (DNMTS) task was performed similarly to the DMTS task. In the DNMTS task, however, the sample visual stimulus was shown again along with a novel alternative, which the monkey needed to choose to obtain the food reward. All monkeys in the control and hTau-overexpressing groups performed the DMTS task preoperatively and the DNMTS task postoperatively.

All experimental procedures were recorded with FHD video cameras.

### Single molecule array (Simoa)-based CSF analysis

To collect CSF samples from the monkeys, a lumbar puncture was performed with a 22 G traumatic needle between L4 and L5. The CSF samples were transferred to sterile low-binding microcentrifuge tubes and then centrifuged at 10,000* g* at 4 °C for 5 min. Next, the total tau, phospho-tau 181, phospho-tau 231, Aβ42, Aβ40, and NfL peptide levels were analysed for all samples using Quanterix’ ultrasensitivity digital biomarker detection technology with the Simoa® HD-X Analyser™ (Quanterix, Billerica, MA, USA), the latest model of the fully automated Simoa bead-based immunoassay platform. The assay kits used in this study were the Simoa® Neurology 3-Plex A Kit (101,995, Quanterix, USA), the Simoa™ pTau-181 V2 Advantage Kit (103,714, Quanterix, USA), the Simoa™ pTau-231 Advantage Kit (102,292, Quanterix, USA), and the Simoa™ NF-light V2 Advantage kit (104,073, Quanterix, USA).

### Nissl staining

Nissl staining was performed with a Nissl Stain Kit (cresyl violet method, Solarbio, G1430). Within this kit, reagent A is a cresyl violet staining solution, and reagent B is a Nissl differentiation solution. First, the brain tissue was placed in reagent A and incubated at 56 °C for 1 h. Then, the brain tissue was rinsed with deionized water. Next, the brain tissue was placed in reagent B for 1 min (this step was repeated until the tissue background was nearly colourless). Then, the brain tissue was rapidly dehydrated with anhydrous ethanol and cleared with xylene. Finally, the brain tissue was sealed with resinene.

### Statistical analysis

Image processing was performed using the 64-bit versions of ImageJ (National Institutes of Health, USA) and ZEN 2.5 (Carl Zeiss, Germany). The 3D reconstructions and tissue volumes were obtained using Brainsight software (Rogue Research, Montréal, Québec, Canada).

All statistical analyses were performed with GraphPad Prism v8.4.2 (GraphPad Software, La Jolla, CA, USA), and datasets were assessed for normality parameters prior to the significance tests. Statistical significance was assessed using two-tailed Student’s *t* test when comparing two groups. When analysing multiple groups, we used a one-way ANOVA with Tukey’s post hoc test to determine the statistical significance. For the variables measured longitudinally at several time points, we analysed the data using repeated measures ANOVA with Tukey’s post hoc test. A *p* value < 0.05 was considered significant.

Data are presented as the mean ± SEM, and data were recorded and analysed blindly, whenever possible.

## Results

### Establishment of an NHP model with AD-like pathology through hippocampal overexpression of tau

**Fig. 1:**
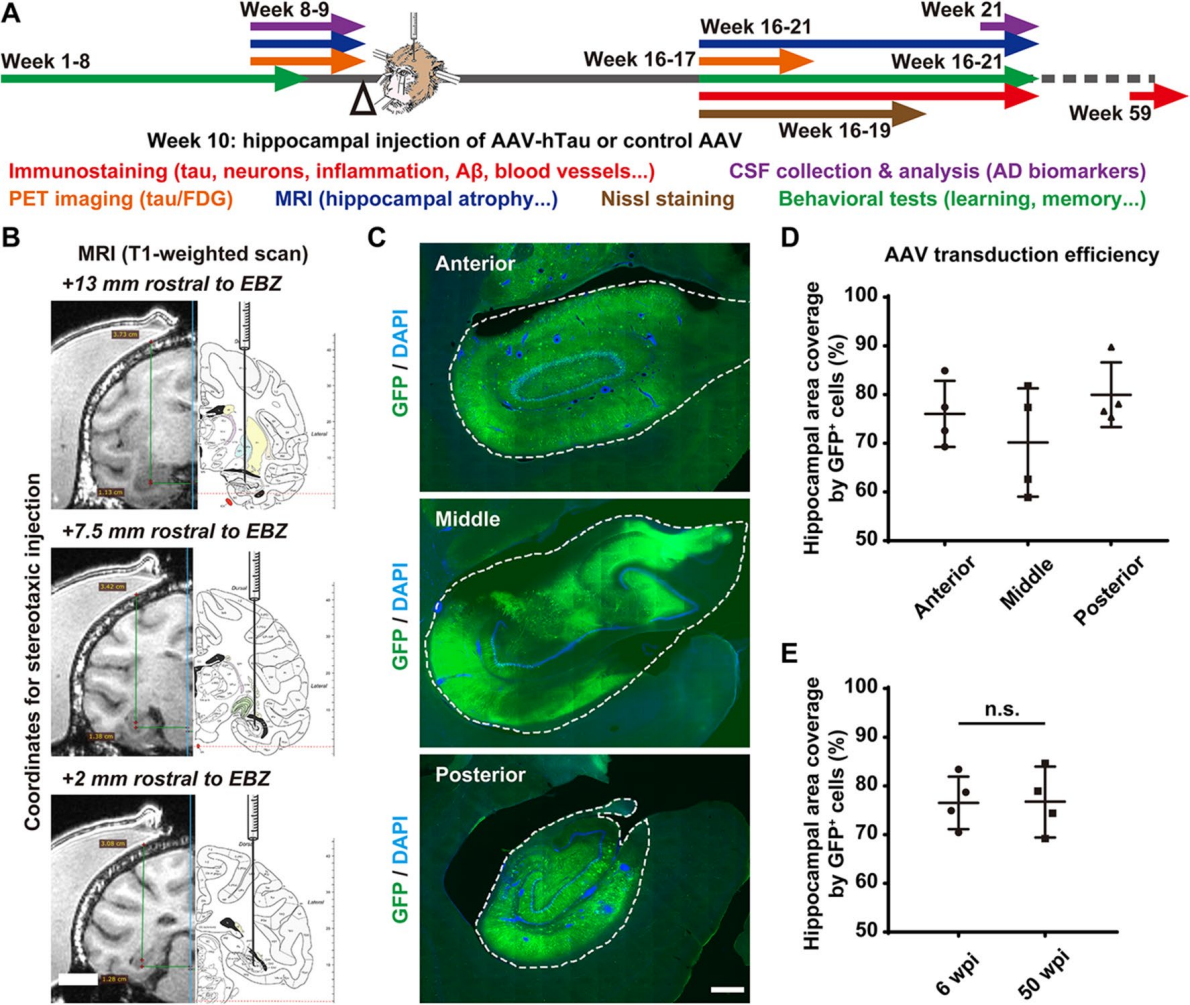
Construction of an NHP model with AD-like pathology through stereotactic AAV injection into bilateral hippocampi.** A** Schematic diagram depicting the construction and evaluation process of the rhesus monkey model with AD-like pathology. The stereotactic injection of AAVs was performed between week 9 and week 10. The monkeys are evaluated for changes in tau expression/phosphorylation, neuronal survival, neuroinflammation, blood vessels, hippocampal volume, CSF biomarkers for AD and cognitive functions before and after viral infection. The NHP model construction and initial evaluation process took approximately 21 weeks. **B** The T1-weighted MRI scans and coronal atlas of the rhesus monkey brain illustrate the coordinates for stereotaxic injection of AAVs. Six sets of coordinates were determined for each individual animal before viral injection based on 3D reconstruction of the monkey brain from T1-weighted MRI scans. EBZ, ear-bar zero. **C** Representative images of GFP immunostaining show the AAV-induced broad expression of GFP (exogenous gene) throughout the monkey hippocampus 8 weeks after viral injection. The dashed lines outline the edges of the hippocampi. Scale bar, 1 mm. **D,E** Quantifications of the average transduction efficiencies of the AAVs in terms of hippocampal area coverage by GFP.^+^ cells in different regions of the hippocampus (**D**) or at different time points after viral injection (**E**). “n.s.” means “not (statistically) significant” (*P* > 0.05). One-way ANOVA with Tukey’s post hoc test (**D**). Student’s *t* test (**E**). *N* = 4

To develop a tauopathy-induced NHP model with AD-like pathology, we employed 18 adult rhesus monkeys (7–15 years old, 16 males and 2 females, Table 1) and selected the hippocampus as the site of hTau overexpression since it is closely related to AD [25–27]. Under monitored anaesthesia, we injected either hTau-expressing AAVs (AAV Syn::FLPo + AAV CAG::FRT-hTau) into the bilateral hippocampi of 11 rhesus monkeys or control AAV (AAV CAG::FRT-hTau) into the bilateral hippocampi of 7 rhesus monkeys (Table 1). After 6–12 weeks or 50 weeks following viral injection, we evaluated these monkeys for changes in tau levels, tau phosphorylation, neuronal populations, neuroinflammation, and blood vessels with immunostaining and Nissl staining. We also tested these monkeys for changes in tau levels, hippocampal mass, CSF indices including AD biomarkers, MRI/PET indices of cognitive function, and behavioural parameters before and after model construction (Fig. 1A, Table 1).

For each monkey, six sets of coordinates for stereotaxic injection of AAVs were predetermined based on the T1-weighted MRI scan of its brain collected using a customized head coil for macaques (Medcoil MK80, Suzhou, China, Fig. 1B). After a single round of AAV injection performed at 6 evenly distributed injection sites, AAV vectors (Syn::GFP) were efficiently delivered to a major portion of the hippocampus, judging by the AAV-induced broad expression of GFP throughout the hippocampus (Fig. 1C). The mean transduction efficiency of the AAVs regarding hippocampal area coverage by GFP^+^ cells was ~ 75% across different regions of the hippocampus (Fig. 1D), with a temporal profile that was stable from 6 to 50 weeks after viral injection (Fig. 1E), indicating broad and long-lasting expression of exogenous genes throughout the monkey hippocampus.

### Identification of high levels of tau expression throughout virus-injected NHP brains

**Fig. 2:**
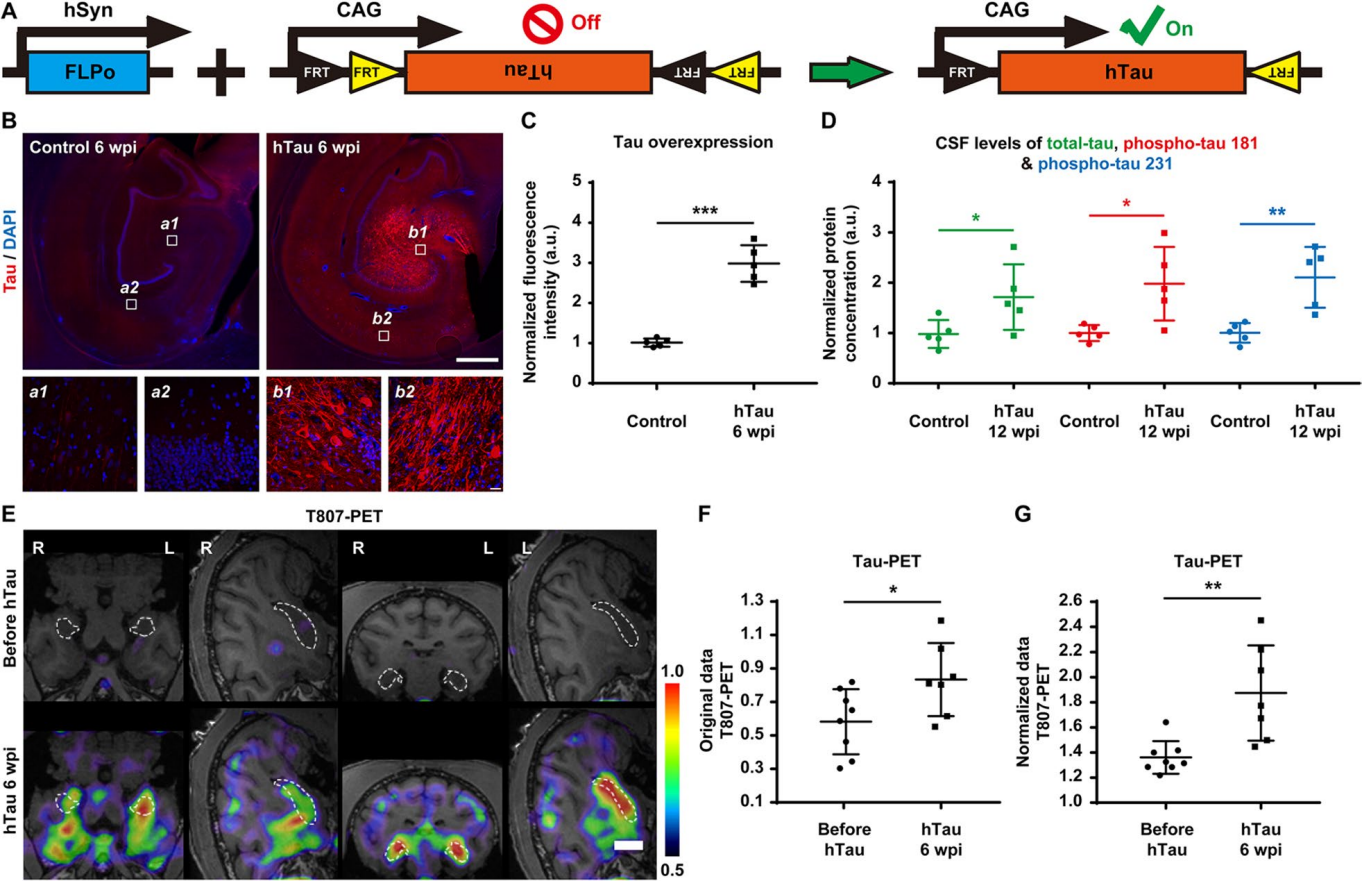
High levels of tau expression throughout the monkey hippocampus after AAV injection.** A** Schematic diagram of our engineered AAV constructs (Syn::FLPo and CAG::FRT-hTau) used to express FLPo recombinase, which in turn will activate the expression of hTau. **B** Representative images of tau immunostaining demonstrate the AAV-induced overexpression of tau in neurons within the monkey hippocampus 6 weeks after viral injection. Compared to that of endogenous tau (left), the expression level of tau in the hTau-overexpressing monkey brains (right) was significantly higher, especially in neuronal somas and apical dendrites. Insets show higher magnification of the CA3 (a1, b1) and CA1 (a2, b2) regions. Scale bar, 1 mm (top) and 20 mm (inset, bottom). **C** Quantification of the normalized fluorescence intensity of tau staining in the monkey brains. The expression levels of tau in the monkey hippocampus after 6 weeks of hTau overexpression were significantly higher than those of endogenous tau in the control monkey hippocampus. ***P < 0.001. Student’s *t* test. *N* = 5. **D** Quantification of the normalized total tau (green), phospho-tau 181 (red), and phospho-tau 231 (blue) concentrations in monkey CSF determined by Simoa-based biomarker analysis. The CSF levels of total tau, phospho-tau 181, and phospho-tau 231 were significantly higher in the hTau-overexpressing monkeys 12 weeks after AAV injection. *P < 0.05, **P < 0.01. Student’s *t* test. *N* = 5. **E** Representative ^18^F-T807 PET/MRI fusion images indicate higher tau expression in the monkey hippocampus 6 weeks after AAV injection. The linear colour scale with the standardized uptake value (SUV) range is shown in the right part of the figure. The dashed lines outline the edges of the hippocampi. Scale bar, 1 cm. **F,G** Quantifications of hippocampal.^18^F-T807 retention (**F**, original SUV values; **G**, hippocampus-to-cerebellum SUV ratios) after 6 weeks of hTau overexpression. *P < 0.05; **P < 0.01. Student’s *t* test. *N* = 7

The overexpression of hTau was achieved by delivering a pair of AAV vectors (Syn::FLPo and CAG::FRT-hTau) into the monkey hippocampus. These AAVs overexpress site-specific recombinase FLPo in neurons under the synapsin promoter and then induce robust hTau expression under the strong ubiquitous CAG promoter via FRT recombination (Fig. 2A). To assess tau expression levels in the virus-injected hippocampus, we performed tau immunostaining on monkey brain slices collected 6 and 10 weeks after AAV injection. The expression of tau in the hippocampus of the monkeys injected with AAV Syn::FLPo and AAV CAG::FRT-hTau was significantly higher than that in the control monkeys (Fig. 2B, top row), and excessive tau accumulation was evident in both the somas and the apical dendrites of many hippocampal neurons (Fig. 2B, bottom row and Additional Fig. 1). Quantitatively, hippocampal tau levels at 6 weeks after viral injection in experimental monkeys were ~ threefold those in the control monkeys (Fig. 2C).

Along with total tau, phospho-tau 181 and phospho-tau 231 in plasma or blood correlate positively with the disease severity of AD and hence can serve as a specific biomarker for AD [28–33]. Therefore, we applied the Simoa technique to measure the concentrations of total tau, phospho-tau 181, and phospho-tau 231 in monkey CSF samples gathered before and after 12 weeks of hTau overexpression (Fig. 2D). As expected, the levels of total tau, phospho-tau 181, and phospho-tau 231 in monkey CSF samples were markedly elevated 12 weeks after AAV injection, mimicking the characteristics of AD.

Recently, the radiotracer ^18^F-T807 has been widely used for PET scanning of the brain to help identify the presence and estimate the distribution of tau pathology, a distinctive characteristic of AD. Distinct from the traditional immunostaining approach, ^18^F-T807 PET imaging can be employed to repeatedly measure tau levels in the brain of the same animal over the course of weeks or even months [34–36]. Consistent with the immunostaining and CSF analysis results, the ^18^F-T807 PET scan also exhibited significant tau accumulation in the monkey hippocampus 6 weeks after AAV injection (Fig. 2E). Both the original values of hippocampal ^18^F-T807 retention and the hippocampus-to-cerebellum ^18^F-T807 retention ratios increased considerably after 6 weeks of hTau overexpression (Fig. 2F,G).

More interestingly, the overexpression of tau could be detected throughout the monkey cortex 50 weeks after viral injection (Additional Fig. 2). This brain-wide spreading of tau pathology was possibly achieved through tau propagation using prion-like mechanisms [23, 37].

### Accumulation of 3R/4R tau isoforms and hyperphosphorylated tau in the virus-injected NHP brain

The tau isoforms in mammalian brains can be classified into 3R or 4R tau depending on how many microtubule binding repeats they contain. Based on the dominant isoforms appearing in the tau aggregates, tauopathies can be subdivided into 3R, 4R, and 3R/4R mixed tauopathies. As a 3R/4R mixed tauopathy, AD involves both 3R and 4R tau isoforms [38–40]. In contrast to human brains, adult rodent brains do not express the 3R tau isoform [3, 4], which makes it difficult to study isoform profiles of tau pathology in AD in rodent models.

**Fig. 3:**
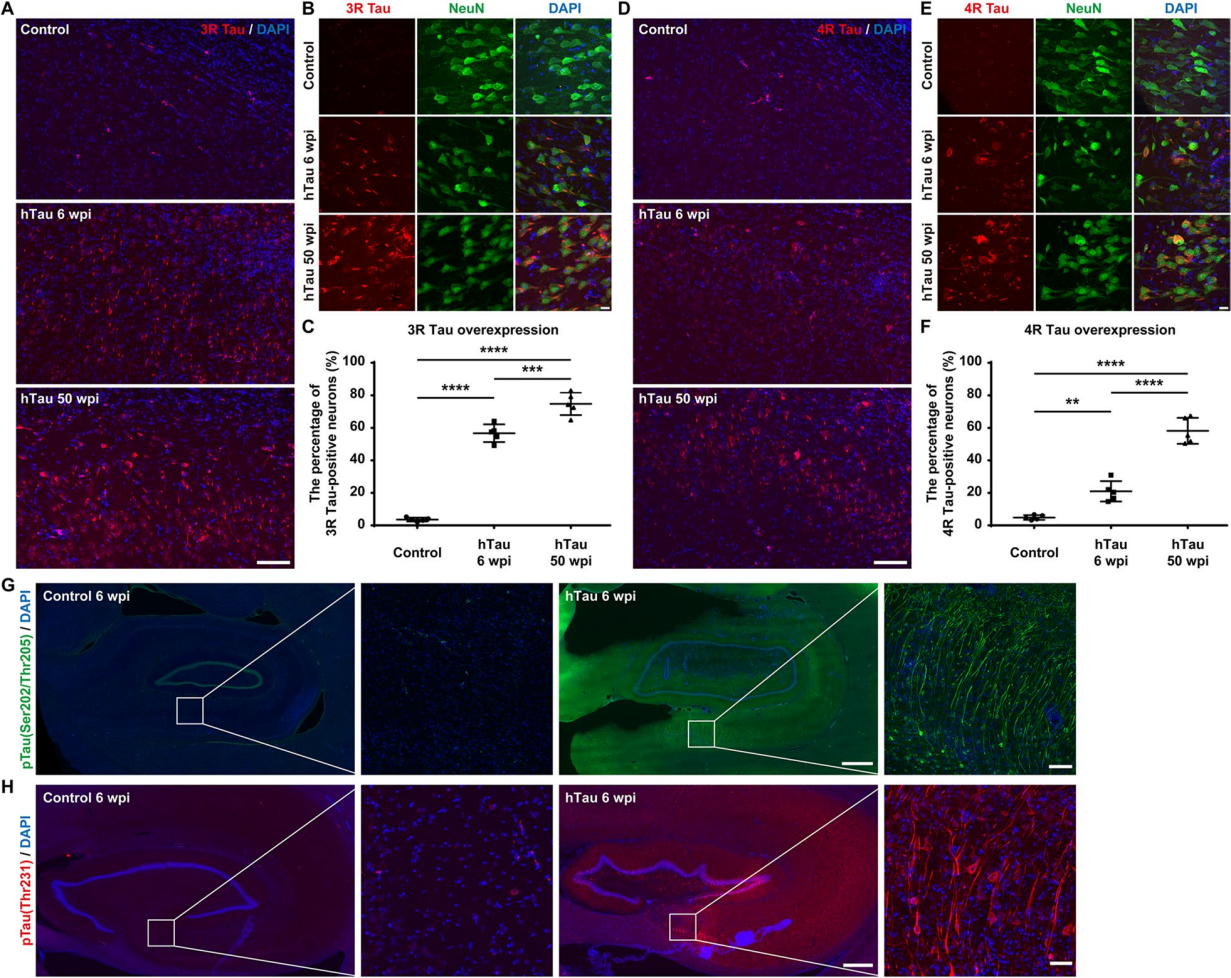
3R and 4R tau accumulation and tau hyperphosphorylation in the monkey hippocampus after hTau overexpression.** A** Representative images of 3R tau immunostaining demonstrate the accumulation of 3R tau within the monkey hippocampus. Compared to the accumulation of endogenous 3R tau (top), the accumulation of 3R tau increased significantly after 6 weeks (middle) and 50 weeks (bottom) of hTau overexpression. Scale bar, 100 mm. **B** Representative images of 3R tau and NeuN costaining show the accumulation of 3R tau in neuronal somas and neurites in the monkey hippocampus. Scale bar, 10 mm. **C** Quantifications of the percentage of 3R tau-positive neurons within all the NeuN^+^ cells in the monkey hippocampus. ***P < 0.001; ****P < 0.0001. One-way ANOVA with Tukey’s post hoc test. *N* = 5. **D** Representative images of 4R tau immunostaining demonstrate the accumulation of 4R tau within the monkey hippocampus. Compared to the accumulation of endogenous 4R tau (top), the accumulation of 4R tau increased significantly after 6 weeks (middle) and 50 weeks (bottom) of hTau overexpression. Scale bar, 100 mm. **E** Representative images of 4R tau and NeuN costaining show the accumulation of 4R tau in neuronal somas and neurites in the monkey hippocampus. Scale bar, 10 mm. **F** Quantifications of the percentage of 4R tau-positive neurons within all the NeuN.^+^ cells in the monkey hippocampus. Note the dramatic increase in the percentage of 3R/4R tau-positive neurons after 6 and 50 weeks of hTau overexpression. **P < 0.01; ****P < 0.0001. One-way ANOVA with Tukey’s post hoc test. *N* = 5. **G** Representative images of phospho-tau (Ser202, Thr205) immunostaining demonstrate the dramatic increase in phospho-tau (Ser202, Thr205) in the monkey hippocampus after 6 weeks of AAV-induced tau overexpression. Insets show higher magnification of the CA1 regions. Scale bar, 1 mm (left) and 20 mm (right, inset). **H** Representative images of phospho-tau (Thr231) immunostaining show the robust increase in phospho-tau (Thr231) in the monkey hippocampus after 6 weeks of AAV-induced tau overexpression. These results suggest the accumulation of paired helical filaments (PHFs, the building blocks of NFTs). Insets show higher magnification of the CA4 regions. Scale bar, 500 mm (left) and 10 mm (right, inset)

The monkey brain slices were immunostained with antibodies against 3R/4R tau isoforms after 6 and 50 weeks of tau overexpression. Widespread 3R and 4R tau accumulation was detected in neurons throughout the hippocampi of monkeys injected with hTau-expressing AAVs, whereas spontaneous 3R and 4R tau accumulation was very low in the hippocampi of control group monkeys (Fig. 3A, B, D, E). Quantitatively, 3R tau-positive neurons represented approximately 50 ~ 60% and 70 ~ 80% of the NeuN^+^ neurons in the monkey hippocampus 6 and 50 weeks after AAV injection, respectively (Fig. 3C). 4R tau-positive neurons represented approximately 20 ~ 30% and 50 ~ 70% of NeuN^+^ neurons in the monkey hippocampus 6 and 50 weeks after AAV injection, respectively (Fig. 3F). Therefore, our AD model NHPs accumulate both 3R and 4R tau isoforms in the brain; thus, the tauopathies observed in our AD model NHPs resemble those observed in AD patients.

The hyperphosphorylation of tau leads to the formation of NFTs in AD. More specifically, the phosphorylation at S202 and T205 located in a proline-rich region of the tau molecule can enhance tau polymerization and induce tau filament formation [41, 42]. In contrast, the phosphorylation at T231 can be detected in the initial stages of preclinical AD, when it hinders microtubule assembly and disrupts microtubule dynamic stability [43, 44].

Here, we labelled pre- and mature NFTs using a phospho-tau (S202/T205) antibody (AT8). Unexpectedly, after only 6 weeks of tau overexpression, many hippocampal neurons already exhibited strong phospho-tau (S202/T205) staining in both somata and neuronal processes, indicating rapid induction of NFT formation in the brains of these monkeys (Fig. 3G). Similarly, we tried to detect tau hyperphosphorylation and eventual NFT formation by immunostaining with a phospho-tau (T231) antibody. The robust phospho-tau (T231) staining in the somata and neurites of many hippocampal neurons 6 weeks after AAV injection suggested that our tau overexpression strategy successfully induced hyperphosphorylated tau as well as tau tangles in the monkey hippocampus (Fig. 3H).

### Significant neural degeneration induced by tau overexpression

**Fig. 4:**
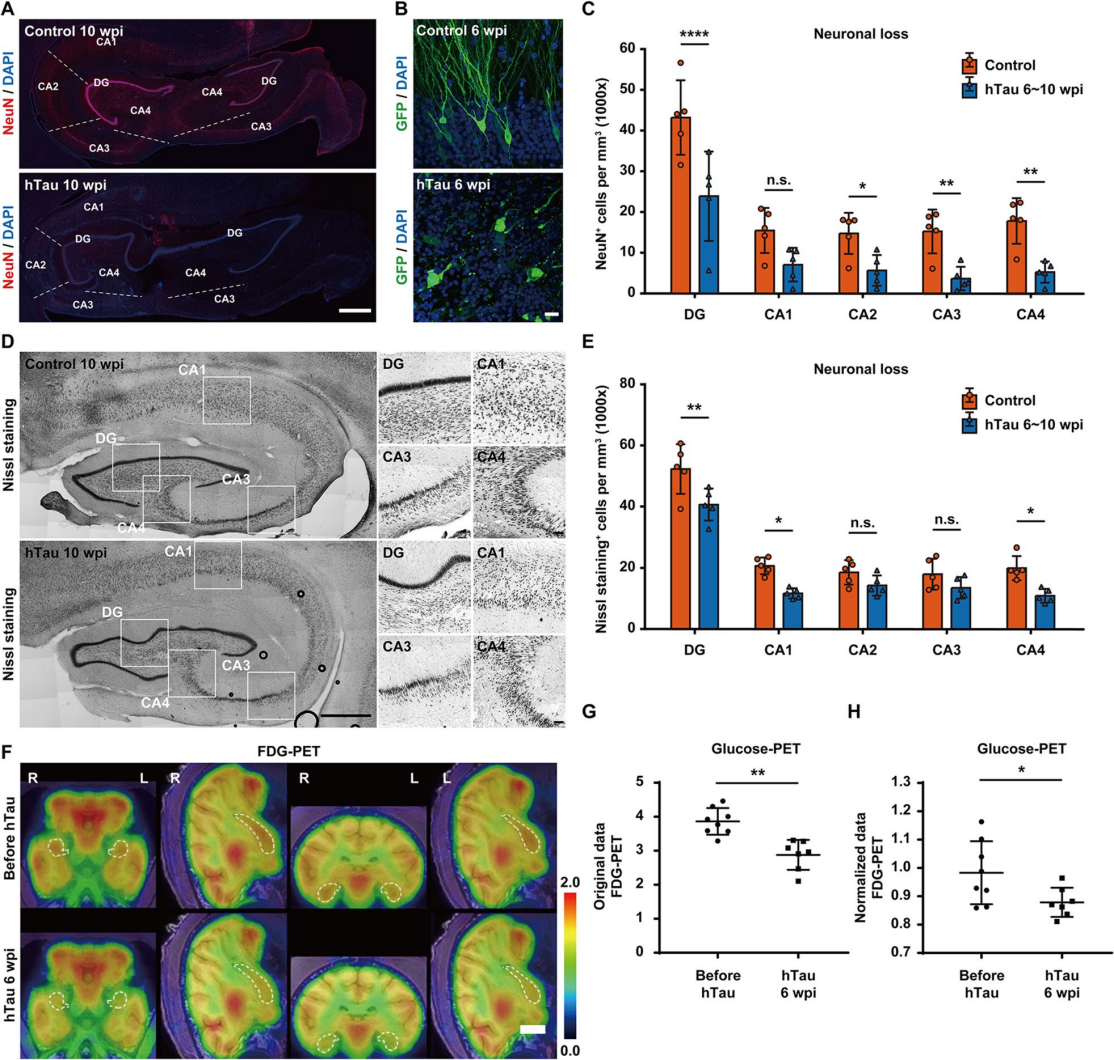
Dramatic neurodegeneration and neuronal loss in the monkey hippocampus after hTau overexpression.** A** Representative images of NeuN immunostaining indicate neurodegeneration and neuronal loss within the monkey hippocampus. The number of NeuN^+^ cells decreased significantly in the monkey hippocampus after 10 weeks of hTau overexpression. The dashed lines separate neighbouring regions of the hippocampus. Scale bar, 1 mm. **B** Representative images of GFP immunostaining show marked neuritic dystrophy and neuronal damage in the monkey hippocampus after 6 weeks of hTau overexpression. Scale bar, 20 mm. **C** Quantification of the number of NeuN^+^ cells per mm^3^ in each hippocampal region of monkeys without hTau overexpression (orange) or with 6 ~ 10 weeks of hTau overexpression (blue). *P < 0.05; **P < 0.01; ****P < 0.0001. “n.s.” means “not (statistically) significant” (*P* > 0.05). One-way ANOVA with Tukey’s post hoc test. *N* = 5. **D** Representative images of Nissl staining suggest neurodegeneration and neuronal loss within the monkey hippocampus. The number of Nissl-stained cells decreased in several regions of the monkey hippocampus after 10 weeks of hTau overexpression. Insets show higher magnification of the DG, CA1, CA3, and CA4 regions. Scale bar, 1 mm (left) and 100 mm (right, inset). **E** Quantification of the number of Nissl-stained cells per mm^3^ in each hippocampal region of monkeys without hTau overexpression (orange) or with 6 ~ 10 weeks of hTau overexpression (blue). *P < 0.05; **P < 0.01. “n.s.” means “not (statistically) significant” (*P* > 0.05). One-way ANOVA with Tukey’s post hoc test. *N* = 5. **F** Representative ^18^F-FDG PET/MRI fusion images show decreased hippocampal glucose metabolism, suggesting pronounced neuronal degeneration or death in the monkey hippocampus 6 weeks after AAV injection. The linear colour scale with the standardized uptake value (SUV) range is shown in the right part of the figure. The dashed lines outline the edges of the hippocampi. Scale bar, 1 cm. **G,H** Quantifications of hippocampal.^18^F-FDG retention (**G**, original SUV values; **H**, hippocampus-to-cerebellum SUV ratios) after 6 weeks of hTau overexpression. *P < 0.05; **P < 0.01. Student’s *t* test. *N* = 7

Progressive hippocampal neurodegeneration is a prominent pathological feature of AD and strongly correlates with cognitive impairment in AD patients [45, 46]. Therefore, we labelled the nuclei of postmitotic neurons with NeuN in monkey brain slices after hTau overexpression. Compared to the control group, we observed a substantial loss of NeuN immunoreactivity across the monkey hippocampus after 6 and 10 weeks of hTau overexpression (Fig. 4A, Additional Fig. 3). Considering that reduced NeuN immunoreactivity can be caused by not only neuronal loss but also the depletion of the protein or loss of its antigenicity [47], we analysed the morphology of the neurons in monkey hippocampi with and without tau overexpression. Six weeks after viral injection, the neurons in the hippocampi of monkeys injected with control and GFP-expressing AAVs exhibited the typical morphology of pyramidal neurons, whereas the same group of neurons from monkeys injected with hTau- and GFP-expressing AAVs showed degenerative features with disintegrated cellular structures (Fig. 4B), suggesting that weak NeuN labelling is indeed closely correlated with neuronal degeneration. Quantitatively, the densities of NeuN^+^ cells in the hippocampal DG/CA2/CA3/CA4 regions declined by approximately 50 ~ 65% at 6 ~ 10 weeks after hTau overexpression (Fig. 4C).

To quantify the neuronal population decline induced by tau overexpression, we conducted Nissl staining (cresyl violet) and measured the expression of pan-neuronal markers Tuj1 (β-III-tubulin) and microtubule-associated protein 2 (MAP2) in monkey hippocampi with and without tau overexpression. The neuronal densities in the hippocampi of monkeys injected with hTau-expressing AAVs were significantly lower than the neuronal densities in the hippocampi of monkeys injected with control AAV 10 weeks after viral injection (Fig. 4D). Quantitatively, the densities of Nissl-stained cells in the hippocampal DG/CA1/CA4 regions decreased by approximately 20 ~ 40% after 6 ~ 10 weeks of hTau overexpression (Fig. 4E). Similarly, we also observed a significant loss of Tuj1 and MAP2 immunoreactivity in the somata and neurites of monkey hippocampal neurons after 6 weeks of hTau overexpression (Additional Fig. 4).

FDG-PET brain imaging is a very sensitive and powerful tool to detect subtle glucose metabolic changes in the brain, and hypometabolism in certain brain regions highly correlates with the pathological diagnosis of AD [48–50]. Consistent with the immuno- and Nissl staining results, the metabolic rate of glucose in the monkey hippocampus decreased significantly after 6 weeks of hTau overexpression (Fig. 4F). Both the original values of hippocampal ^18^F-FDG retention and the hippocampus-to-cerebellum ^18^F-FDG retention ratios dropped substantially after 6 weeks of hTau overexpression, indicating marked neuronal degeneration throughout the monkey hippocampus (Fig. 4G,H).

### Hippocampal atrophy following tau overexpression

**Fig. 5:**
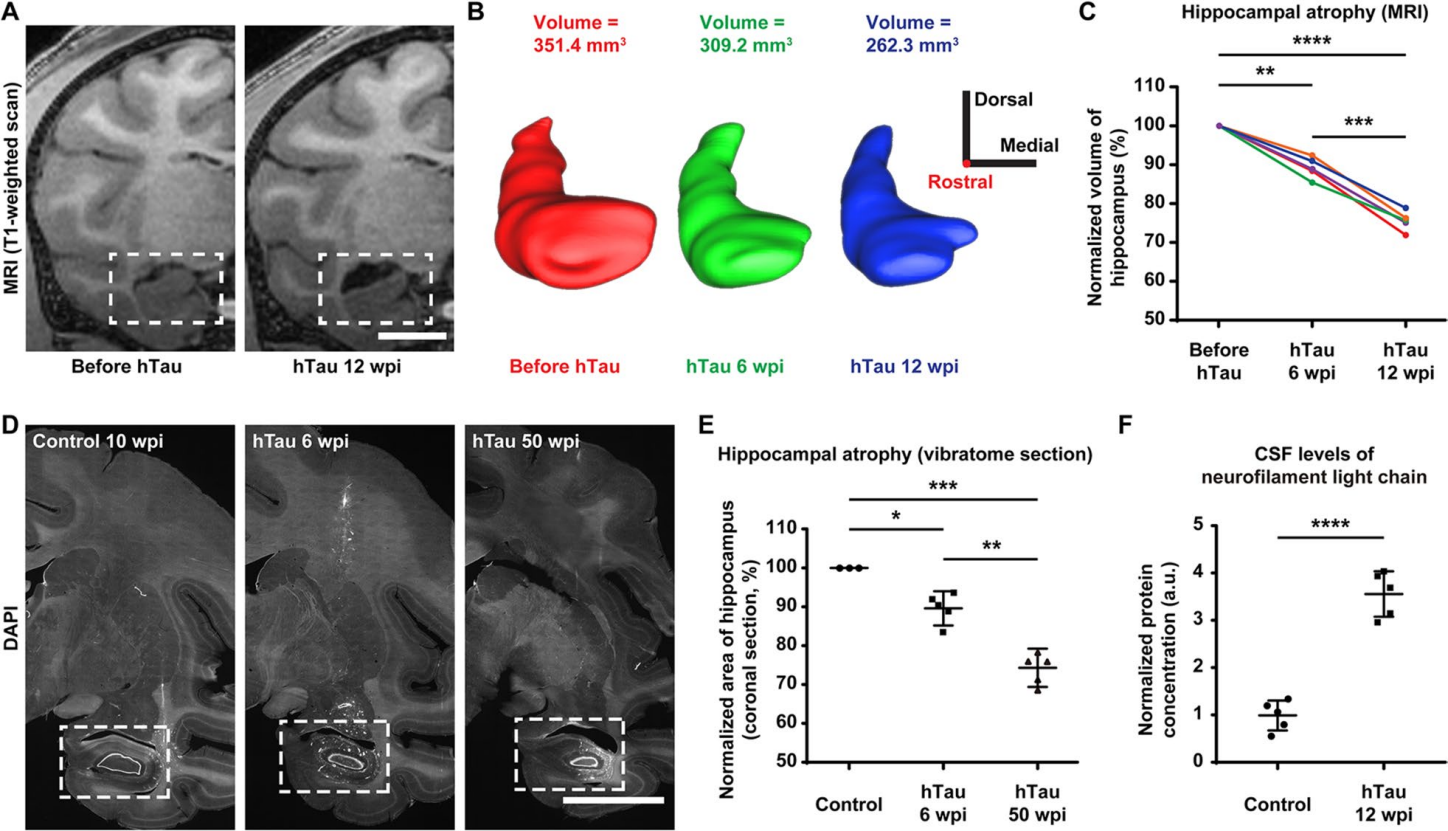
Substantial and progressive hippocampal atrophy after hTau overexpression.** A** Representative coronal T1-weighted MRI images demonstrate hippocampal atrophy in monkey brains after 12 weeks of hTau overexpression. The hippocampal area measured in the coronal section shrinks significantly in the monkey brain 12 weeks after viral injection (right). The dashed boxes highlight the hippocampal areas. Scale bar, 1 cm. **B** Three-dimensional surface reconstructions and the volumes of the right hippocampus of a monkey at 3 different time points throughout hTau overexpression (red, before AAV injection; green, 6 weeks after injection; blue, 12 weeks after injection). This longitudinal study clearly reveals the progressive hippocampal volume decrease after hTau overexpression. The 3D reconstructions and tissue volumes were obtained using Brainsight software (Rogue Research). 3D scale bar, 1 cm in each direction; the rostral-caudal axis is perpendicular to the screen with the rostral direction pointing out of the screen. **C** Quantifications of the longitudinal changes in the normalized hippocampal volumes defined by MRI scans. Each colour represents one animal. **P < 0.01; ***P < 0.001; ****P < 0.0001. Repeated measures ANOVA with Tukey’s post hoc test. *N* = 5. **D** Representative images of coronal vibratome sections (DAPI labelling) showing hippocampal atrophy in monkey brains after 6 and 50 weeks of hTau overexpression. The hippocampal area measured in the coronal section shrinks significantly in the monkey brain 6 (middle) and 50 (right) weeks after viral injection. The dashed boxes highlight the hippocampal areas. Scale bar, 1 cm.** E** Quantifications of the longitudinal changes in the normalized hippocampal areas measured in coronal vibratome sections. *P < 0.05; **P < 0.01; ***P < 0.001. One-way ANOVA with Tukey’s post hoc test. *N* = 5. **F** Quantification of neurofilament light chain (NfL) concentrations in monkey CSF measured by Simoa-based biomarker analysis. The normalized NfL level in CSF was ~ 3.5 times higher in hTau-overexpressing monkeys 12 weeks after AAV injection. ****P < 0.0001. Student’s *t* test. *N* = 5

Reduced hippocampal volume can lead to profound amnesia, which is a core feature of AD. Furthermore, bilateral hippocampal atrophy is associated with an increased risk for the progression of mild cognitive impairment (MCI) to AD [27, 51, 52]. Therefore, we tried to determine whether the monkey hippocampal volume changed after hTau overexpression. We utilized a T1-weighted MRI brain scan to monitor the volume change in the hippocampus after hTau overexpression and observed significant shrinkage of the hippocampus in the same monkey brain 12 weeks after viral injection (Fig. 5A).

By employing the Brainsight neuronavigation system, we reconstructed MRI sections of the monkey hippocampi into 3D models and measured the volumes at different time points after hTau overexpression. The results showed a progressive decrease in the hippocampal volume at 6 and 12 weeks after AAV injection, suggesting tauopathy-induced hippocampal atrophy (Fig. 5B). The quantification of normalized hippocampal volumes measured from the MRI scans revealed an approximately 10% decline at 6 weeks and a 22% decline at 12 weeks after hTau overexpression (Fig. 5C).

To corroborate the MRI analysis findings, we further analysed the hippocampal volume by comparing the cross-sectional areas of the hippocampi in coronal vibratome sections of the monkey brains before and after hTau overexpression. Such a comparison revealed considerable and simultaneous hippocampal shrinkage and lateral ventricle enlargement at both 6 and 50 weeks after viral injection (Fig. 5D). In terms of the extent of this hippocampal atrophy, the normalized hippocampal areas calculated from the coronal vibratome sections decreased by approximately 10% after 6 weeks and 25% after 50 weeks of hTau overexpression (Fig. 5E). Therefore, tau overexpression results in significant hippocampal atrophy.

Furthermore, levels of CSF or plasma neurofilament light chain (NfL) are strongly associated with the progression of AD, and NfL concentration in CSF can reliably predict brain atrophy and cognition in AD [53–55]. Since hippocampal atrophy typically follows elevated CSF NfL concentration, we therefore measured the CSF NfL levels of the monkeys with Simoa technique before and after 12 weeks of hTau overexpression (Fig. 5F). The normalized NfL concentrations of NHPs after 12 weeks of hTau overexpression were more than 3 times higher than those in the control NHPs, suggesting significant AD-like neurodegeneration and neuronal loss, accompanied by notable hippocampal atrophy in these AD model NHPs (Fig. 5F).

### Neuroinflammation induced by tau overexpression

In AD, astrocytes become reactive (activated), and reactive astrocytes are often found in close association with Aβ plaques in the brains of AD patients. Concomitant reactive gliosis is involved in excessive neuroinflammation, oxidative stress, and neuronal dysfunction in AD. Such astrocyte activation can be either harmful due to the loss of neurotrophic effects and the overproduction of proinflammatory molecules or helpful because of the attenuation of Aβ plaque growth and reduction in dystrophic neurites [56–59].

**Fig. 6:**
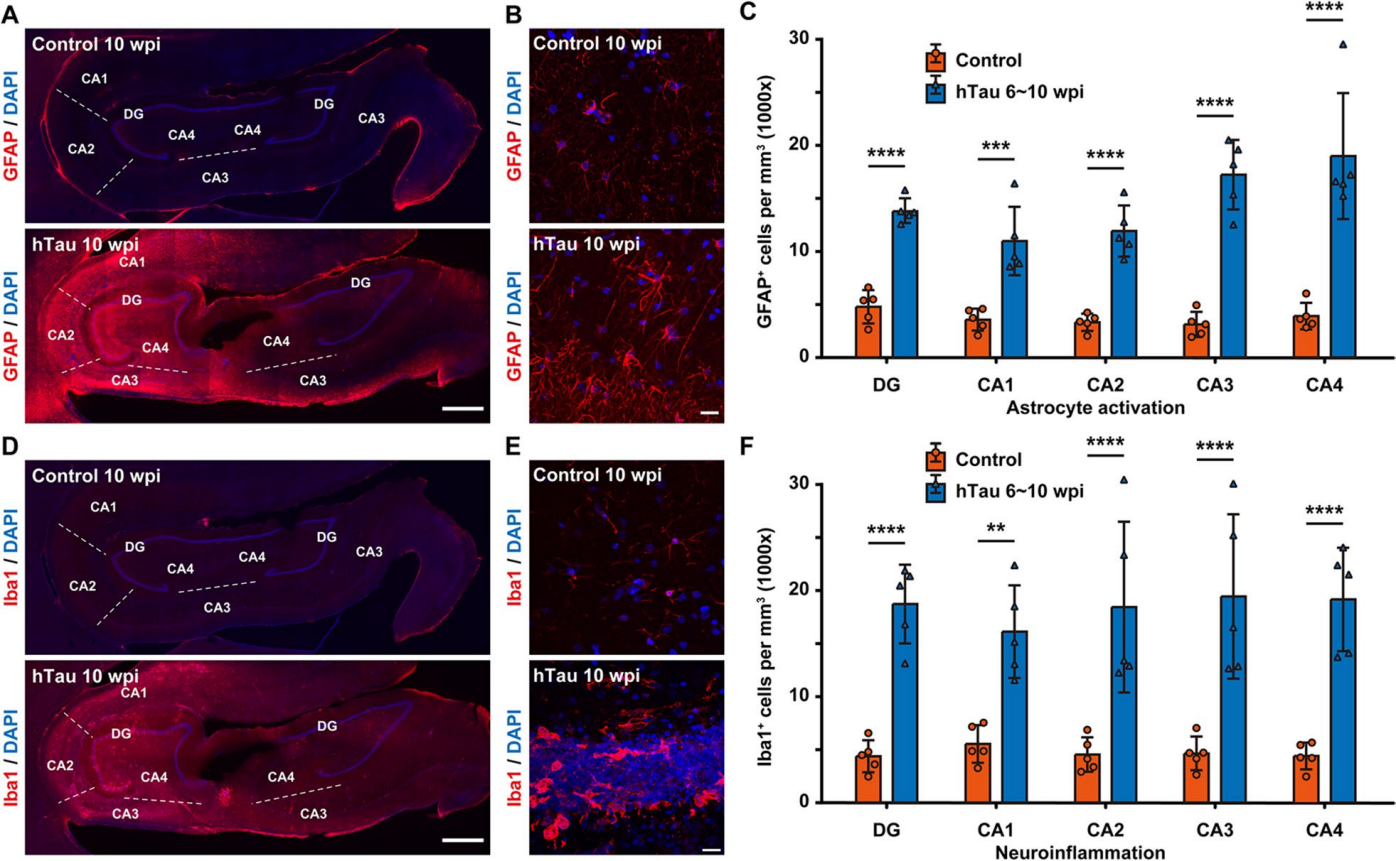
Extensive astrocyte activation and neuroinflammation in the monkey hippocampus after hTau overexpression.** A** Representative images of GFAP immunostaining demonstrate astrocytic activation within the monkey hippocampus. The number of GFAP^+^ cells increased significantly in the monkey hippocampus after 10 weeks of hTau overexpression. The dashed lines separate neighbouring regions of the hippocampus. Scale bar, 1 mm. **B** Representative images of GFAP immunostaining showing the transition of astrocytes from a resting state to a reactive state in the monkey hippocampus after 10 weeks of hTau overexpression. Scale bar, 20 mm. **C** Quantification of the number of GFAP^+^ cells per mm^3^ in each hippocampal region of monkeys without (orange) or with 6 ~ 10 weeks (blue) of hTau overexpression. ***P < 0.001; ****P < 0.0001. One-way ANOVA with Tukey’s post hoc test. *N* = 5. **D** Representative images of Iba1 immunostaining suggest microglial activation and a local neuroinflammatory response within the monkey hippocampus. The number of Iba1^+^ cells increased significantly in the monkey hippocampus after 10 weeks of hTau overexpression. The dashed lines separate neighbouring regions of the hippocampus. Scale bar, 1 mm.** E** Representative images of Iba1 immunostaining show the transition of microglia from a highly ramified resting state to a less ramified, amoeboid reactive state in the monkey hippocampus after 10 weeks of hTau overexpression. Scale bar, 20 mm.** F** Quantification of the number of Iba1^+^ cells per mm.^3^ in each hippocampal region of monkeys without (orange) or with 6 ~ 10 weeks (blue) of hTau overexpression. **P < 0.01; ****P < 0.0001. One-way ANOVA with Tukey’s post hoc test. *N* = 5

We conducted immunostaining of GFAP, a hallmark of astrocytes, and discovered a substantial increase in the GFAP signal across the monkey hippocampus after hTau overexpression (Fig. 6A, Additional Fig. 5). Many GFAP^+^ cells in the hippocampus showed typical morphological changes seen in reactive astrocytes, such as cytoskeletal hypertrophy and outgrowth of particularly long processes (Fig. 6B). Quantitatively, hTau overexpression increased the density of GFAP^+^ cells by 2.5- to fourfold in each region of the monkey hippocampus (Fig. 6C).

Microglial activation and neuroinflammation are considered prominent factors contributing to the pathogenesis of AD. Genome-wide association studies have also demonstrated that many AD risk genes are highly expressed in microglia. Activated microglia can protect against AD by phagocytosing Aβ and cellular debris; alternatively, they can aggravate AD by mediating synapse loss, exacerbating tau phosphorylation, impacting CNS homeostasis and secreting inflammatory factors [60–63].

We performed immunostaining against Iba1, whose expression is upregulated in activated microglia, and we observed a marked increase in Iba1 immunoreactivity across the monkey hippocampus after hTau overexpression (Fig. 6D and Additional Fig. 6). In addition, many Iba1^+^ cells in the hippocampus transformed from having a ramified, star-shaped morphology into having small, spherical, rod-shaped, or amoeboid-like transformed morphologies (Fig. 6E). Moreover, the density of Iba1^+^ cells increased by 2.5- to 3.5-fold in each hippocampal region of the monkeys after hTau overexpression (Fig. 6F). Given that Iba1 is also expressed by resting microglia, whereas CD68 is commonly considered a marker of activated phagocytic microglia since it labels their lysosomes [64], we also carried out immunostaining against CD68 in the hippocampi of hTau-expressing monkeys. Elevated CD68 signals across the hippocampus further confirmed robust immune activation and neuroinflammation throughout the hippocampus of the AD model NHPs (Additional Fig. 7). Collectively, these results indicate that tau overexpression can induce astrocytic and microglial pathologies in the monkey hippocampus.

These reactive astrocytes and activated microglia may produce nitric oxide and inflammatory cytokines (such as interleukins, TNF-α, and TGF-β) that could contribute to a reinforced neuroinflammation cascade, which may amplify the initial neurotoxic insults triggered by tau overexpression to drive neurodegeneration and further brain pathologies of AD. Thus, our results appear to imply the participation of glial cells in the pathogenesis of our AD-like NHP model.

### Decreased clearance and increased accumulation of Aβ after tau overexpression

**Fig. 7:**
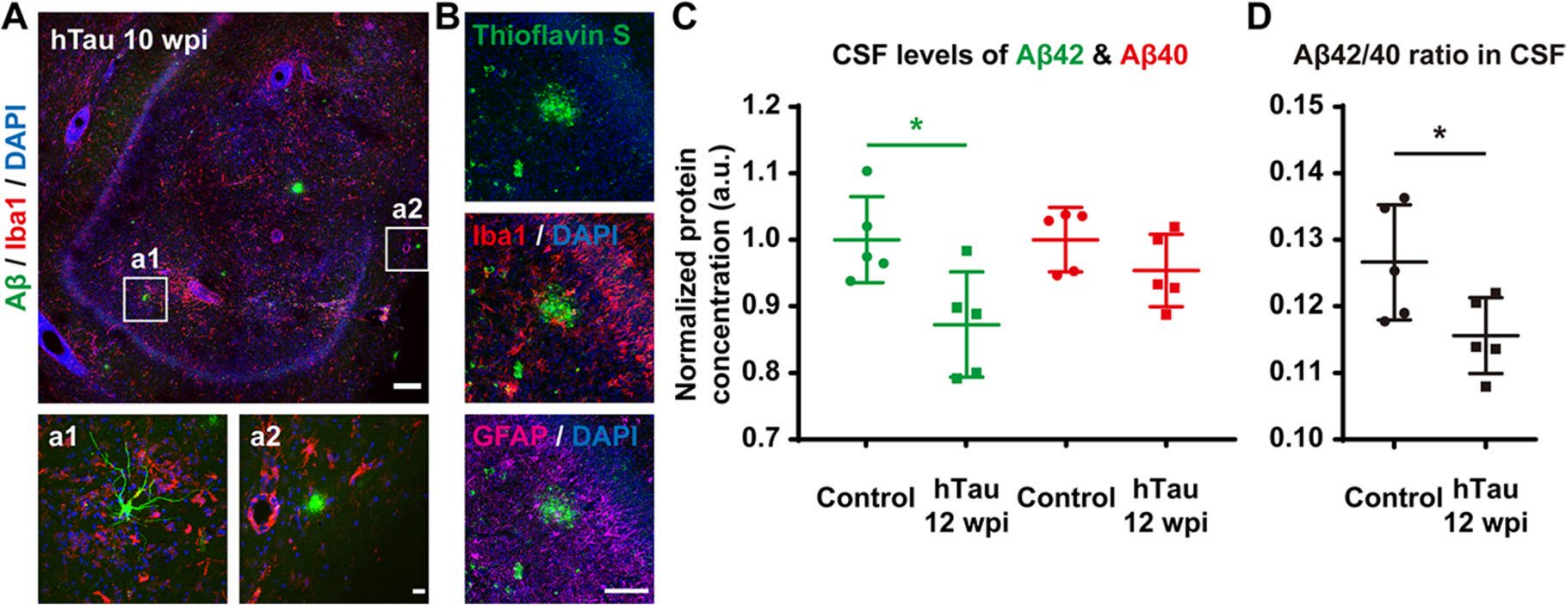
Decreased clearance and increased accumulation of Aβ throughout the monkey hippocampus after hTau overexpression.** A** Representative images of Aβ and Iba1 immunostaining suggest the development of Aβ pathology, including intracellular Aβ oligomers and extracellular Aβ plaques, within the monkey hippocampus 10 weeks after viral injection. Higher magnification insets show intracellular accumulation of soluble Aβ (a1) and an extracellular plaque-like structure of Aβ aggregation (a2). Note the Iba1^+^ microglia surrounding the Aβ-accumulating cells and plaques. Scale bar, 200 mm (top) and 20 mm (inset, bottom). **B** Representative images of Thioflavin-S staining labelling fibrillar Aβ deposits indicate the formation of AD Aβ plaques in the monkey hippocampus 10 weeks after AAV injection. Note the Iba1^+^ microglia and GFAP.^+^ reactive astrocytes gathering around the Aβ plaque. Scale bar, 100 mm. **C,D** Quantification of Aβ42 (**C**, green) and Aβ40 (**C**, red) peptide concentrations, as well as the Aβ42/Aβ40 ratio (**D**) in monkey CSF determined by Simoa-based biomarker analysis. The Aβ42 level and Aβ42/Aβ40 ratio in CSF were significantly higher in hTau-overexpressing monkeys 12 weeks after AAV injection. *P < 0.05. Student’s *t* test. *N* = 5

Along with tauopathy, the production and deposition of Aβ is also a key feature of AD, and diverse findings suggest synergistic effects between Aβ and tau pathologies [65, 66]. Furthermore, most patients with MCI or AD have reduced concentrations of Aβ peptide in the CSF, and a lower CSF or plasma Aβ42/Aβ40 ratio has been considered an indicator of a greater risk of MCI or AD [67, 68]. After 10 weeks of hTau overexpression, we performed immunostaining against Aβ and detected some intracellular Aβ accumulation and extracellular Aβ plaque-like structures within many hippocampal slices of the monkey brains (Fig. 7A). In addition, thioflavin-S staining also revealed scattered Aβ plaque-like deposits surrounded by reactive microglia and reactive astroglia in many hippocampal slices of the monkey brains (Fig. 7B). Moreover, it is worth mentioning that the total quantity of the Aβ load was relatively small, possibly due to the relatively long period needed to develop Aβ pathologies throughout the brain. More interestingly, Simoa-based biomarker analysis showed that the CSF Aβ42 peptide level and Aβ42/Aβ40 ratio dropped considerably after 12 weeks of hTau overexpression (Fig. 7C-D), suggesting a diminished capacity for Aβ clearance in these monkeys with AD-like pathology.

Taken together, the Aβ pathology in these AD-like monkeys may be attributed to either increased Aβ accumulation driven by APP- or BACE-containing endosomes trapped by phosphorylated tau on disrupted microtubules [69–71], or decreased Aβ clearance due to impaired perivascular drainage and glymphatic system caused by tau-induced astrocytic AQP4 depolarization [72, 73]. Therefore, our findings appear to challenge the exclusive downstream role of tau in relation to Aβ.

### Vascular abnormality after tau overexpression

**Fig. 8:**
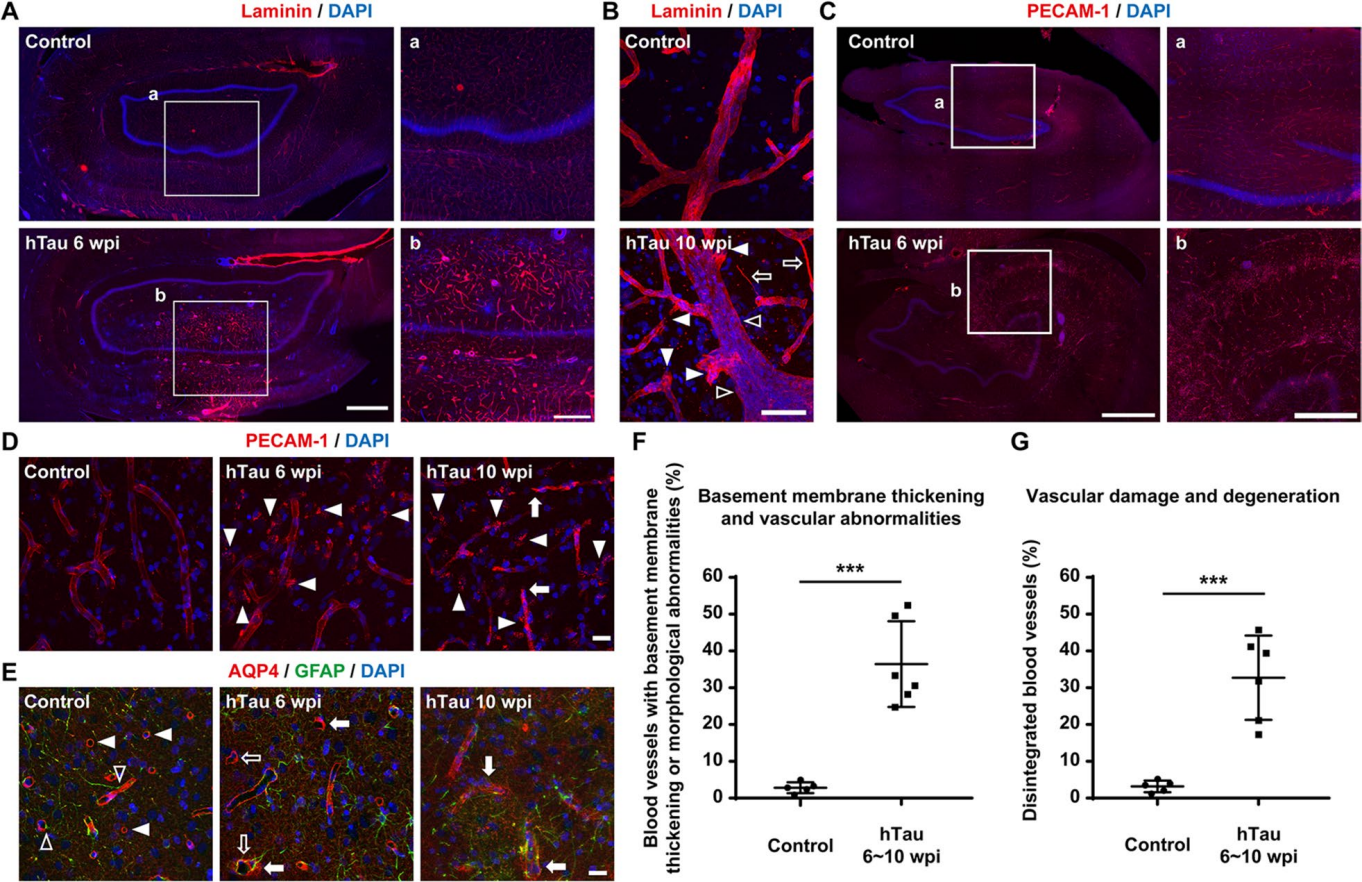
Various vascular abnormalities and damages throughout the monkey hippocampus after hTau overexpression.** A,B** Representative images of laminin immunostaining indicate vascular basement membrane thickening and abnormal vascular morphology alterations in the monkey hippocampus after hTau overexpression. Insets show higher magnification images of the blood vessels in the hippocampus (**A**). Many vascular abnormalities, such as string vessels (**B**, open arrows), vessel occlusion (**B**, open arrowheads), tortuosity, stenosis and bulging of the vascular walls (**B**, closed arrowheads), emerged after hTau overexpression. Scale bar, 1 mm (**A**, left), 500 mm (**A**, inset, right) and 50 mm (**B**). **C,D** Representative images of PECAM1 immunostaining suggest vascular degeneration and damage in the monkey hippocampus after hTau overexpression. There were significantly more disintegrated blood vessels in the monkey hippocampus after 6 weeks of hTau overexpression. Insets show higher magnification images of the blood vessels in the hippocampus (**C**). The progressive vascular degeneration is mainly characterized by blood vessel ruptures (**D**, arrows) and disintegration (**D**, arrowheads). Scale bar, 1 mm (**C**, left), 500 mm (**C**, inset, right) and 20 mm (**D**). **E** Representative images of AQP4 and GFAP staining show relatively diffuse AQP4 expression and substantial astrocytic endfeet retraction from blood vessels in the monkey hippocampus after hTau overexpression. Normally, astrocytic endfeet wrap blood vessels (open arrowheads) with AQP4 water channels on astrocytic endfeet, forming ring-like structures (closed arrowheads). After tau overexpression, AQP4 signals become relatively diffuse (closed arrows), and AQP4.^+^ endfeet detach from the blood vessels (open arrows). Scale bar, 20 mm. **F** Quantification of the percentage of blood vessels with basement membrane thickening or distinct morphological changes in the monkey hippocampus. ***P < 0.001. One-way ANOVA with Tukey’s post hoc test. *N* = 6.** G** Quantification of the percentage of blood vessels with significant structural damage and disintegration in the monkey hippocampus. Note that the percentages of basement membrane thickening, morphologically abnormal, damaged or degenerated blood vessels in the monkey hippocampus after hTau overexpression were significantly higher than the percentages in the control monkey hippocampus. ***P < 0.001. One-way ANOVA with Tukey’s post hoc test. *N* = 6

Considering the clinical and pathological overlap between cerebrovascular disease and AD, many recent studies have shown that vascular dysfunctions, including chronic brain hypoperfusion, blood‒brain barrier leakage, and chronic vascular inflammation, serve as a major trigger in the progression of sporadic AD. In fact, clinicopathological data also indicate an alarming positive feedback loop among vascular, glial, and neuronal dysfunctions dictating the onset and development of AD throughout its entire course [74–77]. Immunostaining against laminin, a main component of the vascular basement membrane (Fig. 8A), in monkey brain slices demonstrated vascular basement membrane thickening after hTau overexpression, which is one of the distinct blood vessel changes observed in AD [78]. Immunostaining against laminin also revealed many vascular abnormalities, such as string vessels (1 ~ 2 μm wide connective tissue strands that are remnants of capillaries but do not carry blood, Fig. 8B, open arrows), tortuous and bulging blood vessels (Fig. 8B, closed arrowheads), and vascular occlusion (Fig. 8B, open arrowheads), after hTau overexpression. Moreover, immunostaining against PECAM-1, a molecule expressed on endothelial and other vascular compartment cells, showed significant vascular damage and degeneration (Fig. 8C) characterized by blood vessel rupture (Fig. 8D, arrows) and disintegration (Fig. 8D, arrowheads) after hTau overexpression. Furthermore, immunostaining for AQP4, a water channel expressed at astrocyte endfeet, revealed alterations in the polarized distribution of AQP4 (Fig. 8E, closed arrowheads and arrows) and astrocytic endfeet retraction from the blood vessels (Fig. 8E, open arrowheads and arrows) after hTau overexpression. In terms of the extent of vascular abnormalities or damage, approximately 35% of the capillaries displayed basement membrane thickening or some abnormal morphological alterations (string vessels/tortuosity and bulging vessels/vascular occlusion) after 6 ~ 10 weeks of hTau overexpression (Fig. 8F). Moreover, approximately 30% of the capillaries were damaged or degenerated after 6 ~ 10 weeks of hTau overexpression (Fig. 8G). Together, these results show that tau overexpression can induce many vascular abnormalities and damage associated with AD in monkey brains.

These vascular abnormalities in monkey brains after tau overexpression add to a growing body of literature on the involvement of tauopathy in the development of vascular pathology in AD. Moreover, this NHP model may become a valuable resource for further research into structural and functional integrity of the BBB in AD.

### Learning and memory deficits after tau overexpression

Cognitive decline, especially memory impairment, is a typical symptom of AD [79, 80]. Therefore, we utilized the Wisconsin General Test Apparatus (WGTA) to perform a “delayed response” (DR) task (Additional video 1) and a “delayed matching-to-sample/delayed nonmatching-to-sample” (DMTS/DNMTS) task (Additional video 2 and 3) to test the spatial working memory and associative learning ability of the rhesus monkeys before and after tau overexpression in bilateral hippocampi [81, 82].

**Fig. 9:**
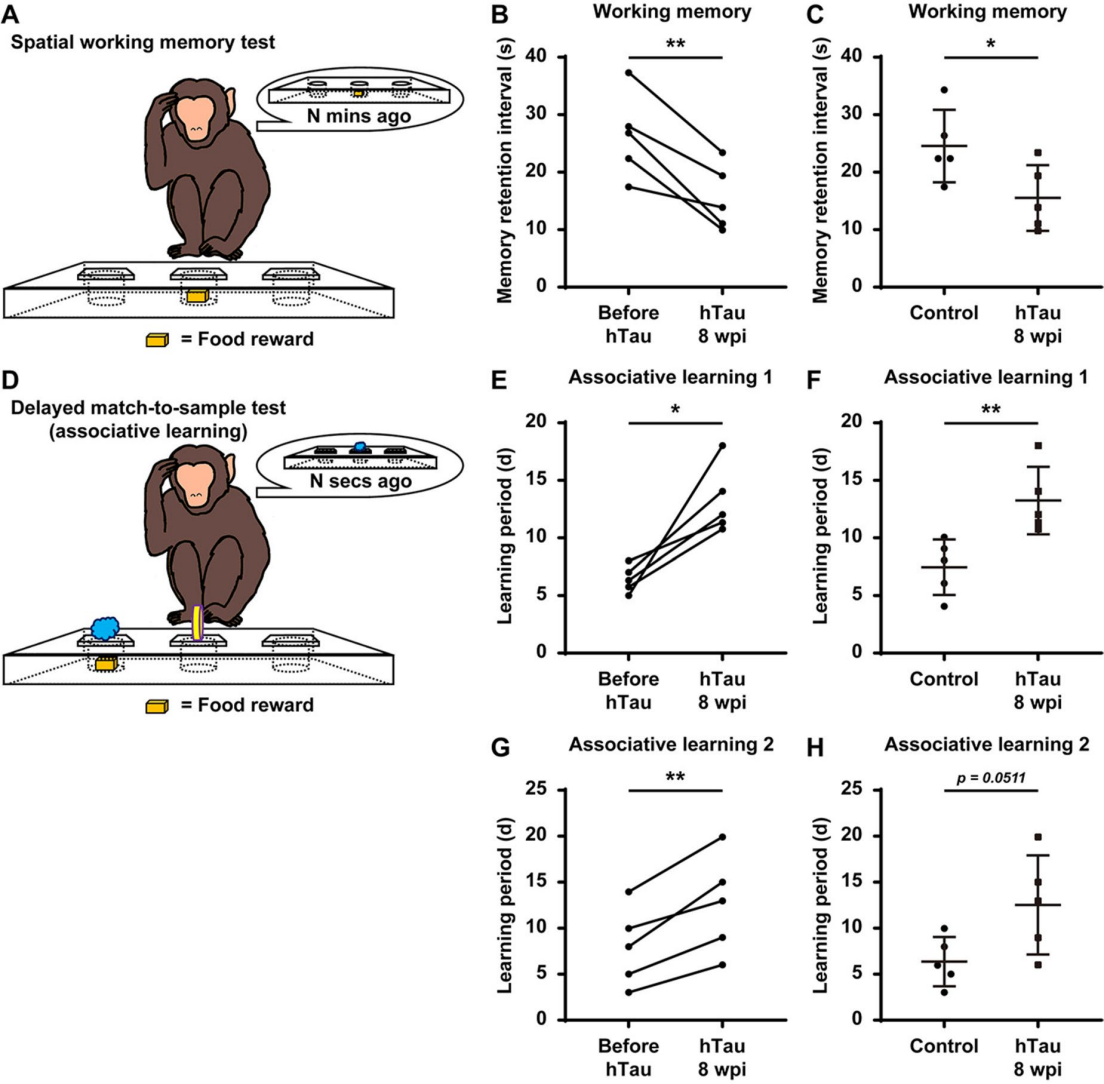
Deteriorated spatial working memory and impaired associative learning ability in monkeys with hTau overexpression.** A** A schematic drawing illustrates the experimental design of the WGTA-based monkey spatial working memory test. **B** Quantification of the memory retention interval before and after hTau overexpression. The working memory capacity of the monkeys declined significantly after hTau overexpression. Each dot represents an individual animal. **P < 0.01. Student’s *t* test. *N* = 5. **C** Quantification of the memory retention interval of the control virus- and hTau-expressing virus-injected monkeys. The working memory capacity of the hTau-expressing virus-injected monkeys was significantly lower than that of the control virus-injected monkeys. Each dot represents an individual animal. *P < 0.05. Student’s *t* test. *N* = 5. **D** A schematic drawing illustrates the experimental design of the WGTA-based monkey delayed match-to-sample test. **E, G** Quantification of the associative learning period before and after hTau overexpression in two associative learning tests (**E**, using katakana characters as visual cues; **G**, using LEGO® blocks as visual cues). Learning ability was noticeably impaired in monkeys after hTau overexpression. Each dot represents an individual animal. *P < 0.05; **P < 0.01. Student’s *t* test. *N* = 5. **F, H** Quantification of the associative learning period of the control virus- and hTau-expressing virus-injected monkeys in two associative learning tests (**F**, using katakana characters as visual cues; **H**, using LEGO.® blocks as visual cues). The learning ability of the hTau-expressing virus-injected monkeys was noticeably weaker than that of the control virus-injected monkeys. Each dot represents an individual animal. **P < 0.01. Student’s *t* test. *N* = 5

In the DR task, the monkeys needed to memorize the position of food rewards in one out of three covered food wells for a short period to successfully retrieve the food reward from the correct well at their first attempt. The maximum period a monkey could reliably hold the position information in its working memory was recorded as its “memory retention interval.” After 8 weeks of hTau overexpression, the monkeys displayed an average decline of 30 ~ 40% in their “memory retention interval,” indicating a robust decline in their spatial working memory capacity (Fig. 9A,B). The monkeys injected with the hTau-expressing AAVs also showed a significant (30 ~ 40%) decrease in their “memory retention interval” compared to the monkeys injected with the control AAV, excluding the possibility that the behavioural decline was due to the aftereffects of the viral injection or the surgery (Fig. 9C).

In the DMTS/DNMTS tasks, the monkeys needed to learn to associate a familiar stimulus (DMTS) or a novel stimulus (DNMTS) with the food reward. The period a monkey required to master these tasks was recorded as its “learning period.” After 8 weeks of hTau overexpression, the monkeys needed a profoundly longer “learning period” to master these tasks, suggesting a significant impairment in associative learning ability in two different DMTS/DNMTS-based associative learning tests (using katakana characters and LEGO® blocks as visual cues). On average, the monkeys would need approximately 50 ~ 80% more training time to acquire the skill to reliably retrieve (success rate ≥ 90%) the food rewards associated with certain visual cues (Fig. 9D, E, G). The monkeys injected with the hTau-expressing AAVs also exhibited a dramatic (70 ~ 100%) prolongation in their “learning period” compared to the monkeys injected with the control AAV, excluding the possibility that these behavioural deficits were due to the aftereffects of the viral injection or the surgery (Fig. 9D, F, H).

Taken together, our findings show that our AD-like NHP model can be generated within a couple of months via a single AAV injection and recapitulates many defining pathological features of AD, including 3R/4R tau accumulation, tau hyperphosphorylation, tau propagation, NFT formation, neuronal loss, hippocampal atrophy, inflammatory responses, Aβ clearance deficit, vascular abnormalities, and multimodal cognitive dysfunctions.

## Discussion

In this study, we established an NHP model of AD with a wide variety of AD-like pathologies ranging from AD-related protein aggregation at the subcellular level to AD-like memory deficits at the system level. The fact that this model can be generated within a few months through a single stereotaxic injection of AAVs may also help our model achieve broader adoption within the biomedical research community.

NHP models of AD are important to bridging the translational gap between the findings from preclinical work in AD rodent models and effective therapies for AD patients. Through the collective efforts of the international community of AD researchers, some spontaneous, nongenetically or genetically induced NHP models of AD with great potential have been developed over the last two decades [8–18, 23, 24]. However, most of these models take a very long period to establish and a substantial amount of cost to generate, rendering it difficult to conduct large-scale evaluations of therapeutic interventions over a practical time frame. In contrast, our NHP model with AD-like pathology can be conveniently produced via a single injection of AAVs into the brains of middle-aged rhesus monkeys within a few months and thus can be broadly adopted by the vast majority of researchers worldwide to identify and analyse potential treatment strategies for AD within a reasonable timeline.

Until the relatively recent detection of p-tau tangles in their entorhinal cortex [70, 83], rhesus macaques were not considered able to develop pathological tauopathy due to a lack of reporting of p-tau accumulation in their brains [84, 85]. Since very few rhesus monkeys live long enough to reach 25 + years of age to show spontaneous tauopathy [70, 83], the induction of p-tau may become highly relevant in the future development of macaque AD models. In fact, leading researchers in the field have already been working on tauopathy induction models in rhesus and cynomolgus monkeys [23, 24]. In addition, rhesus monkeys have rarely shown distinctive neuronal loss in their brains [86] or rapid deterioration of cognitive functions similar to AD patients [87]. Taking all the above into consideration, this study reveals an unexpected yet striking resemblance between our AD model NHPs and AD patients. More specifically, our AD model NHPs not only exhibit widespread 3R/4R tau aggregation, tau hyperphosphorylation, and neuronal loss throughout the hippocampus but also display the multimodal cognitive dysfunctions, neuroinflammation, and microvascular alterations that characterize AD.

Except for a couple of transgenic or genome-edited mouse lines, adult mouse/rat brains lack the 3R tau isoforms that are widely expressed in human patients [88–90]. This is one of the major intrinsic limitations posed by our current rodent models of AD [3, 4]. In contrast to rodents and even marmosets, rhesus monkeys express both 3R/4R tau isoforms in their brains. Hence, we tried to determine whether our NHP model with tau overexpression could reproduce the tau aggregates containing both 4R/3R tau isoforms seen in AD patients. The 3R and 4R tau isoforms increasingly accumulated in many hippocampal neurons from 6 to 50 weeks after AAV injection, and 3R and 4R tau aggregates were clearly present in neuronal somata and processes throughout the monkey hippocampus. Therefore, our NHP model of AD could help not only help explore the mechanisms of the propagation of tauopathies involving both 3R/4R isoforms but also reproduce the complete spectrum of tau pathologies associated with AD, both of which will hopefully lead to the discovery of new therapeutic strategies for AD.

Even though tau is almost exclusively synthesized in and released from neurons, recent studies have suggested vital roles of glial cells and associated neuroinflammation in tauopathy. First, glial cells contribute to the propagation of pathological tau. For instance, microglia mediated tau spreading in mouse brains, and oligodendrocytes transmitted tau aggregates along white matter tracts independent of neuronal tau pathology [91–93]. Second, pathological tau promotes neuroinflammation through microglial and/or astrocyte activation in tau mouse models and human tauopathies. For example, tau fibrils induced microglial inflammation via TLR2 in AD mice, and FTLD-tau was associated with astrocyte reactivity in FTLD-tau patients [94–96]. Third, gliosis and neuroinflammation in response to tau pathology lead to neurodegeneration, as astrocytes and microglia respond to neuronal damage in progressively uncontrolled ways [97, 98]. Moreover, pathological tau exposure triggers astrocyte and microglial senescence in AD mice and patients, and the accumulation of senescent glial cells initiates cognition-associated neuronal loss in AD mice [99, 100]. In our NHP model of AD, the brain-wide tau propagation throughout the cortex at 50 wpi (Additional Fig. 2) is possibly achieved via glial cell-driven spreading of pathological tau; the increased astrocyte and microglial reactivity and extensive neuroinflammation in the hippocampi after tau overexpression (Fig. 6) seem to support the theory that tauopathy induces neuroinflammation through microglial and astrocyte activation; and the substantial neuronal loss and hippocampal atrophy after tau overexpression (Fig. 4, 5) are in line with the notion that tau-evoked gliosis and neuroinflammation lead to significant neurodegeneration. Hence, our results provide new evidences for the involvement of glial cells in the pathogenesis of AD.

Excessive accumulations of Aβ and tau are widely recognized as the defining hallmarks of AD. Furthermore, mounting experimental and clinical evidence seems to point to a copathogenic interaction between Aβ and tau, which jointly accelerates the onset and progression of AD [65, 66]. In line with this Aβ-tau synergetic theory, our AD-like NHP model induced by hTau overexpression in monkey hippocampi did display scattered intracellular Aβ accumulation, occasional parenchymal Aβ plaque-like deposits, and significant Aβ clearance deficits, as revealed by immunostaining and CSF Aβ measurements. However, the distribution of Aβ aggregation in the brains of this NHP model is relatively sparse, which is inconsistent with the high density of senile plaques in many AD patients. Such a discrepancy may be due to the long period that the abundant Aβ plaques need to build up through p-Tau-driven Aβ production or tau-related Aβ1-42 clearance [69, 101, 102]. We are planning to perform long-term measurements of Aβ deposition in the brains of our AD model NHPs to test this hypothesis in future studies.

Aβ was often believed to be upstream of tau in AD development and trigger the hyperphosphorylation and aggregation of tau. However, some recent studies have challenged the theory that tau functions exclusively downstream of Aβ. For instance, large-scale longitudinal studies of human brains showed that tau pathology starts a decade before Aβ plaque formation [103]; the production of Aβ was found to correlate with tau expression levels in mouse CNS and monkey spinal cord [24, 104]; the post-mortem distribution of Aβ plaques suggested Aβ generation and release from axon terminals with tau pathology [105]; ageing rhesus monkey studies indicated that aggregated phosphorylated tau traps APP-transporting endosomes where APP cleavage drives Aβ production [69, 70]; hyperphosphorylated tau increased Aβ generation in axons via microtubule disruption, leading to APP and BACE1 accumulations [71]. Furthermore, deletion of astrocytic AQP4 suppressed soluble Aβ clearance through perivascular drainage system, and loss of perivascular AQP4 polarization impeded glymphatic exchange and escalated Aβ plaque formation in mice [72, 73]. The intracellular Aβ accumulation in the hippocampus of our NHP model (Fig. 7A) is probably due to the “endosomal traffic jams” or microtubule disruption caused by aggregated phosphorylated tau (Fig. 3G, H), whereas the extracellular Aβ plaque-like structures in the hippocampus of our NHP model (Fig. 7B) may result from AQP4 depolarization induced by tau overexpression (Fig. 8E), leading to impaired perivascular drainage and glymphatic clearance of Aβ (Fig. 7C, D). Thus, our findings appear to help establishing a potential cycle between tau and Aβ in driving and spreading AD pathologies.

Pathological studies of the brains of AD patients demonstrated that AD starts in the entorhinal cortex and hippocampus [106, 107], which is the exact reason why we chose to overexpress tau in the hippocampi of rhesus monkeys. However, AD will eventually lead to widespread damage throughout the brain, so an ideal NHP model of AD should display the major pathologic features not only in the hippocampus but also throughout the whole brain. Interestingly, we did detect robust overexpression of tau throughout the cerebral cortex of the monkeys 50 weeks after viral injection (Additional Fig. 2), which can probably be explained by the “prion-like” propagation of tau aggregation [37, 108]. Since mounting evidence suggests that the propagation of tau would cause the dysfunction and degeneration of the neuronal networks [109, 110], we will subsequently examine whether there is significant neuronal loss, neuroinflammation or microvascular abnormalities in all these cortical regions with tau pathology.

The pathological effects of cerebral amyloid angiopathy (CAA, vascular Aβ accumulation) in AD have been thoroughly studied [111], whereas the impact of tau on vascular pathology in AD has only recently started to draw the attention of the scientific community. AAV-mediated overexpression of tau in mouse brain was found to cause vascular defects such as increased capillary thickness and vascular compression by swollen astrocytic endfeet [112]. Overexpressing mutant tau in forebrain neurons in tau transgenic mice was shown to initiate breakdown of the BBB, induce blood vessel abnormalities (atypical and spiraling capillaries…) and alter blood vessel diameter and density [113, 114]. More interestingly, tau oligomers and neurofibrillary tangles in AD patients compromised the integrity of the cerebral microvessels and remodelled the wall of cerebral arteries even before the onset of CAA [115, 116]. Furthermore, tau-induced neuroinflammation can additionally damage BBB, leading to cerebral microvascular damages in human tauopathies [117]. The vascular abnormalities and damages in our NHP model of AD (Fig. 8) may result from perivascular tau accumulation, tau toxicity and neuroinflammation, which may impact vascular architecture, cerebral blood flow and BBB integrity. However, the detailed molecular and cellular mechanisms underlying vascular basement membrane thickening, vascular abnormalities, vascular degeneration and astrocytic endfeet retraction from the blood vessels in our NHP model remain unexplored and warrant further investigation in the future.

Together, our AD-like NHP model with 3R/4R tau aggregation, tau hyperphosphorylation, neuronal loss, hippocampal atrophy, neuroinflammation, Aβ clearance deficits, NFT formation, blood vessel damage and cognitive decline will serve as an effective tool for unveiling the pathogenic mechanisms of AD and developing disease-modifying treatments in the future.

## Conclusions

In this work, we developed an NHP model of AD that can be generated within 2 ~ 3 months through a single injection of hTau-expressing AAVs into bilateral hippocampi of rhesus monkeys. After 2–3 months of tau overexpression, 3R/4R tau aggregates, hyperphosphorylated tau, neurodegeneration, neuronal loss, hippocampal atrophy, neuroinflammation, vascular abnormalities, impaired Aβ clearance, spatial working memory and associative learning deficits were observed in these AD-like NHPs. Our model may potentially be adopted by researchers worldwide, and hence facilitate mechanistic studies and accelerate therapeutic development for AD.

### Supplementary Information

**Additional file 1: Additional figure 1. **Representative images of tau immunostaining demonstrating the AAV-induced overexpression of tau in neurons within the monkey hippocampus 10 weeks after viral injection. Compared to that of endogenous tau (top), the expression level of tau in the hTau-overexpressing monkey brains (bottom) was significantly higher. Scale bar, 1 mm.

**Additional file 2: Additional figure 2. **Representative images of tau immunostaining showing the overexpression of tau throughout the monkey brain 50 weeks after viral injection, indicating the spread of tau pathology from the AAV injection sites to the whole brain, possibly through prion-like propagation. Insets show higher magnification of the hippocampal (a1, b1) and cortical (a2, b2, a3, b3, a4, b4) regions. Scale bar, 1 mm (half brain, left) and 50 mm (inset, bottom right).

**Additional file 3: Additional figure 3. **Representative images of NeuN and tau immunostaining indicating neuronal degeneration and loss in the monkey hippocampus 6 weeks after viral injection. The number of NeuN^+^ cells decreased significantly when hTau expression levels were high. Insets show higher magnification of the CA3 (a1, b1) and CA1 (a2, b2) regions. Scale bar, 1 mm (top) and 20 mm (inset, bottom).

**Additional file 4: Additional figure 4. (A) **Representative images of MAP2 (green) and Tuj1 (red) immunostaining showing neurodegeneration and neuronal loss within the monkey hippocampus. Note that the immunoreactivity of both MAP2 and Tuj1 decreases in the monkey hippocampus after 6 weeks of hTau overexpression. Scale bar, 1 mm. **(B)** Higher magnification images of the regions of interest in the white squares in (A). There was a significant loss of Tuj1 and MAP2 immunoreactivity in the somata and neurites of monkey hippocampal neurons after 6 weeks of hTau overexpression. Scale bar, 50 mm.

**Additional file 5: Additional figure 5. **Representative images of GFAP immunostaining showing astrocytic activation within the monkey hippocampus. The number of GFAP^+^ cells increased significantly in the monkey hippocampus 6 weeks after hTau overexpression. Many GFAP^+^ cells in the hippocampus undergo typical morphological changes seen in reactive astrocytes after hTau overexpression. Insets show higher magnification of the CA3 (a1, b1) and CA1 (a2, b2) regions. Scale bar, 1 mm (top) and 20 mm (inset, bottom).

**Additional file 6: Additional figure 6. **Representative images of Iba1 immunostaining suggesting microglial activation and a neuroinflammatory response in the monkey hippocampus. The number of Iba1^+^ cells increased significantly in the monkey hippocampus after 6 weeks of hTau overexpression. Note that many Iba1^+^ cells in the hippocampus showed typical morphological changes seen in reactive microglia after hTau overexpression. Insets show higher magnification of the CA3 (a1, b1) and CA1 (a2, b2) regions. Scale bar, 1 mm (top) and 20 mm (inset, bottom).

**Additional file 7: Additional figure 7.** Representative images of CD68 immunostaining showing elevated CD68 signals across the hippocampus, further confirming robust immune activation and neuroinflammation throughout the hippocampus after 6 weeks of hTau overexpression. Insets show higher magnification of the CA3 region. Scale bar, 1 mm (top) and 20 mm (inset, bottom).

**Additional file 8: Additional video 1.** WGTA-based monkey spatial working memory test. The delayed response (DR) task was conducted using the Wisconsin General Test Apparatus (WGTA), in which a monkey sat in its home cage in front of a tray that contained 3 food wells covered by identical swing-away lids. Initially, the experimenter baited one of the wells with food in front of the monkey, covered it, and then lowered an opaque screen to block the food tray from the monkey’s view. After a certain delay period, the screen was removed, which allowed the monkey to retrieve the food from the baited food well, which had to be recalled from working memory. Food reinforcers were randomly distributed among 3 wells over 30 trials per day (≥ 26 correct choices for 3 consecutive days to pass), and the length of the delays was gradually increased according to a stepwise procedure as the monkeys demonstrated mastery of the task.

**Additional file 9: Additional video 2.** WGTA-based monkey delayed matching-to-sample test. A delayed matching-to-sample (DMTS) task was also performed with the WGTA. The experimenter first presented a sample visual stimulus (a katakana character or LEGO^®^ block) to the monkey. Following a random interval (up to 5 seconds), the same sample visual stimulus and another visual distractor were both presented to the monkey, and the monkey needed to choose the visual stimulus that matched the sample stimulus to obtain the food reward. Each incorrect choice was followed by a 10-second timeout. The monkeys underwent 30 DMTS trials per day (≥ 26 correct choices for 3 consecutive days to pass), and both the sample visual stimuli and the visual distractors were randomly selected each day from either 46 basic katakana alphabet letters or a large pool of LEGO^®^ blocks (> 200 combinations).

**Additional file 10: Additional video 3.** WGTA-based monkey delayed nonmatching-to-sample test. A delayed nonmatching-to-sample (DMTS) task was also performed with the WGTA. The experimenter first presented a sample visual stimulus (a katakana character or LEGO^®^ block) to the monkey. Following a random interval (up to 5 seconds), the same sample visual stimulus and another visual distractor were both presented to the monkey, and the monkey needed to choose the visual stimulus that was different from the sample stimulus to obtain the food reward. Each incorrect choice was followed by a 10-second timeout. The monkeys took 30 DMTS trials per day (≥ 26 correct choices for 3 consecutive days to pass), and both visual stimuli were randomly selected each day from either 46 basic katakana alphabet letters or a large pool of LEGO^®^ blocks (> 200 combinations).

## Data Availability

The datasets supporting the conclusions of this article are either included within this article (and its additional files) or available from the corresponding author on reasonable request.

## Abbreviations

AD: Alzheimer’s disease
NHPs: Nonhuman primates
CSF: Cerebrospinal fluid
MRI: Magnetic resonance imaging
PET: Positron emission tomography
3R tau: 3-Repeat tau
4R tau: 4-Repeat tau
hTau: Human tau
AAV: Adeno-associated virus
NFT: Neurofibrillary tangle
iDGU: Isopycnic density gradient ultracentrifugation
qPCR: Quantitative PCR
LAL assay: Limulus amoebocyte lysate assay
AAALAC: The Association for Assessment and Accreditation of Laboratory Animal Care
IACUC: The Institutional Animal Care and Use Committee
IHC: Immunohistochemistry
PFA: Paraformaldehyde
2D: 2-Dimensional
3D: 3-Dimensional
ROI: Region of interest
SUV: The standard uptake value
WGTA: The Wisconsin general test apparatus
DR task: The delayed response task
DMTS task: The delayed matching-to-sample task
DNMTS task: The delayed nonmatching-to-sample task
Simoa: Single molecule array
Tuj1: β-III-tubulin
MAP2: Microtubule-associated protein 2
MCI: Mild cognitive impairment
