# Supplementary figures and images for: A nonhuman primate model with Alzheimer’s disease-like pathology induced by hippocampal overexpression of human tau
Source: Alzheimers Res Ther. 2024 Jan 27;16:22. doi: 10.1186/s13195-024-01392-0 (PMC10821564; doi:10.1186/s13195-024-01392-0)

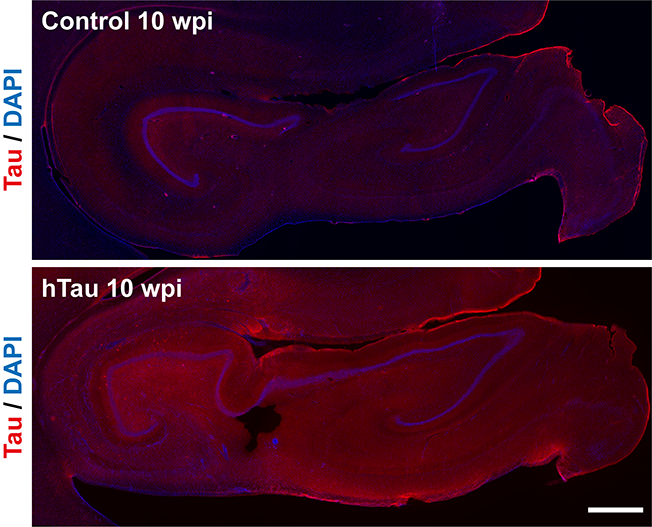

Supplement: Supplementary file 1 — Additional file 1: Additional figure 1. Representative images of tau immunostaining demonstrating the AAV-induced overexpression of tau in neurons within the monkey hippocampus 10 weeks after viral injection. Compared to that of endogenous tau (top), the expression level of tau in the hTau-overexpressing monkey brains (bottom) was significantly higher. Scale bar, 1 mm. [file 13195_2024_1392_MOESM1_ESM.tif]

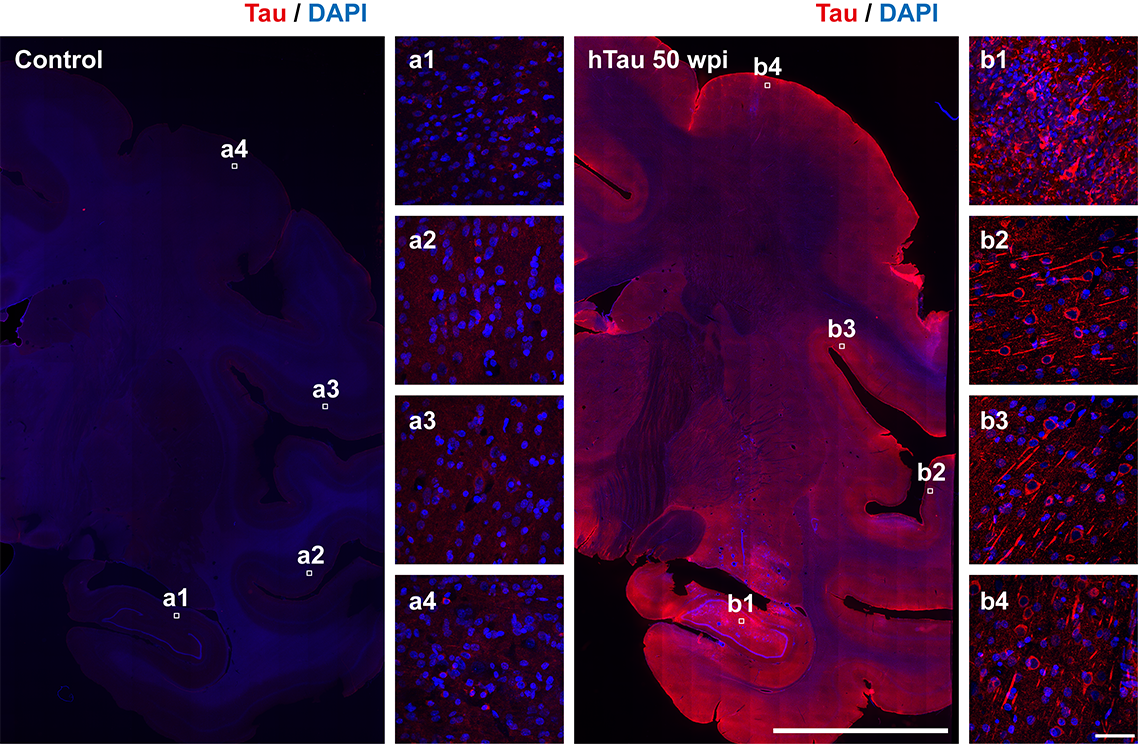

Supplement: Supplementary file 2 — Additional file 2: Additional figure 2. Representative images of tau immunostaining showing the overexpression of tau throughout the monkey brain 50 weeks after viral injection, indicating the spread of tau pathology from the AAV injection sites to the whole brain, possibly through prion-like propagation. Insets show higher magnification of the hippocampal (a1, b1) and cortical (a2, b2, a3, b3, a4, b4) regions. Scale bar, 1 mm (half brain, left) and 50 mm (inset, bottom right). [file 13195_2024_1392_MOESM2_ESM.tif]

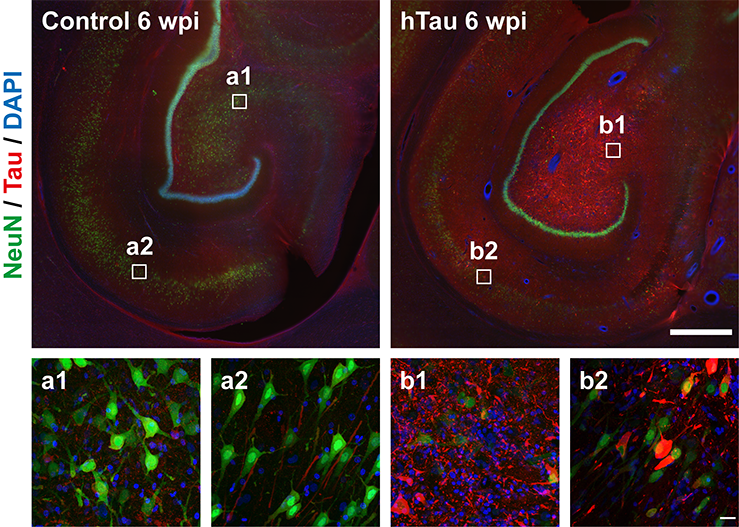

Supplement: Supplementary file 3 — Additional file 3: Additional figure 3. Representative images of NeuN and tau immunostaining indicating neuronal degeneration and loss in the monkey hippocampus 6 weeks after viral injection. The number of NeuN+ cells decreased significantly when hTau expression levels were high. Insets show higher magnification of the CA3 (a1, b1) and CA1 (a2, b2) regions. Scale bar, 1 mm (top) and 20 mm (inset, bottom). [file 13195_2024_1392_MOESM3_ESM.tif]

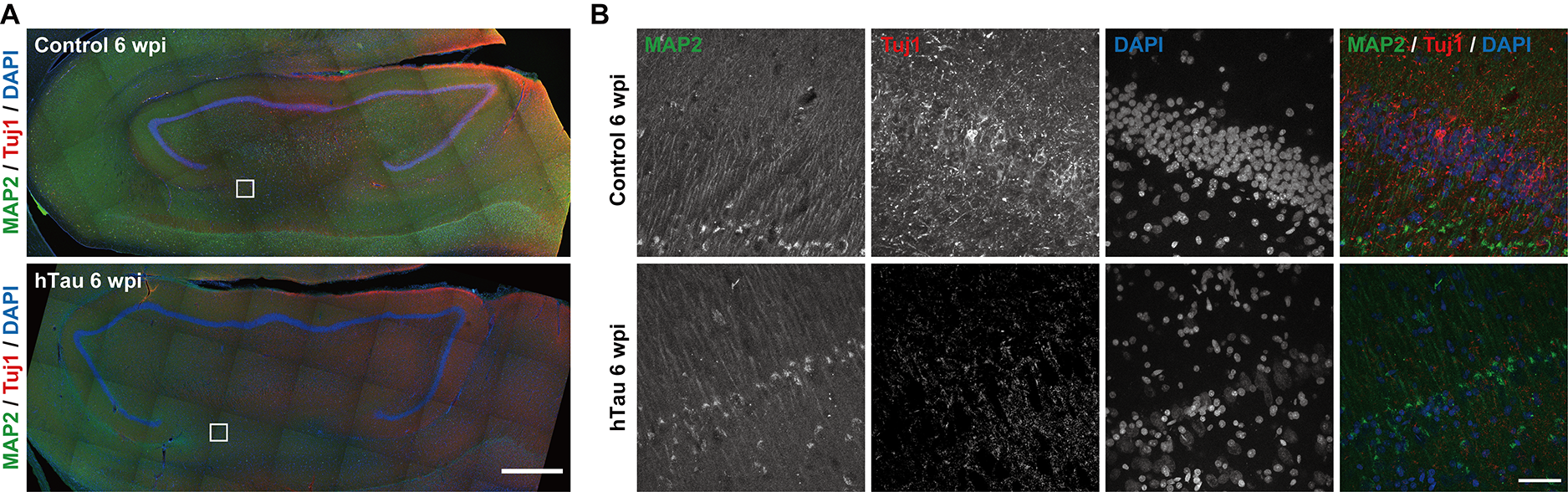

Supplement: Supplementary file 4 — Additional file 4: Additional figure 4. (A) Representative images of MAP2 (green) and Tuj1 (red) immunostaining showing neurodegeneration and neuronal loss within the monkey hippocampus. Note that the immunoreactivity of both MAP2 and Tuj1 decreases in the monkey hippocampus after 6 weeks of hTau overexpression. Scale bar, 1 mm. (B) Higher magnification images of the regions of interest in the white squares in (A). There was a significant loss of Tuj1 and MAP2 immunoreactivity in the somata and neurites of monkey hippocampal neurons after 6 weeks of hTau overexpression. Scale bar, 50 mm. [file 13195_2024_1392_MOESM4_ESM.tif]

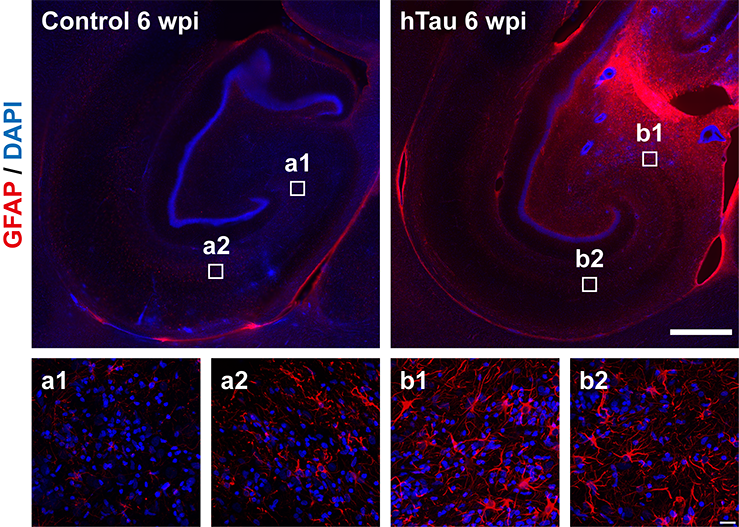

Supplement: Supplementary file 5 — Additional file 5: Additional figure 5. Representative images of GFAP immunostaining showing astrocytic activation within the monkey hippocampus. The number of GFAP+ cells increased significantly in the monkey hippocampus 6 weeks after hTau overexpression. Many GFAP+ cells in the hippocampus undergo typical morphological changes seen in reactive astrocytes after hTau overexpression. Insets show higher magnification of the CA3 (a1, b1) and CA1 (a2, b2) regions. Scale bar, 1 mm (top) and 20 mm (inset, bottom). [file 13195_2024_1392_MOESM5_ESM.tif]

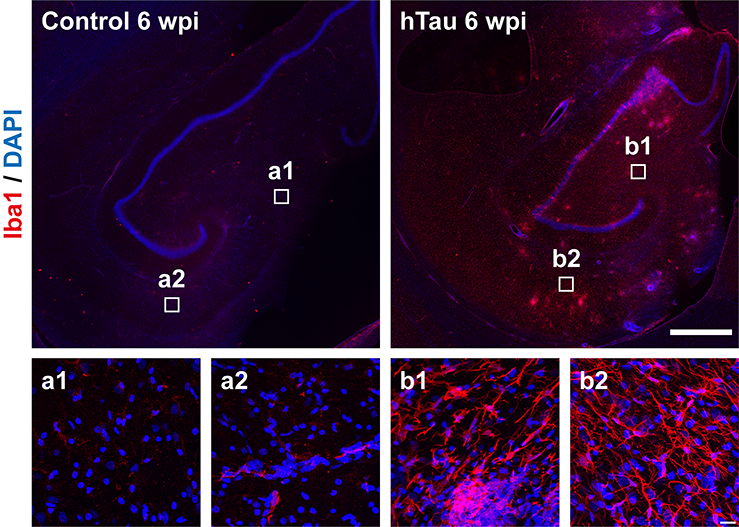

Supplement: Supplementary file 6 — Additional file 6: Additional figure 6. Representative images of Iba1 immunostaining suggesting microglial activation and a neuroinflammatory response in the monkey hippocampus. The number of Iba1+ cells increased significantly in the monkey hippocampus after 6 weeks of hTau overexpression. Note that many Iba1+ cells in the hippocampus showed typical morphological changes seen in reactive microglia after hTau overexpression. Insets show higher magnification of the CA3 (a1, b1) and CA1 (a2, b2) regions. Scale bar, 1 mm (top) and 20 mm (inset, bottom). [file 13195_2024_1392_MOESM6_ESM.tif]

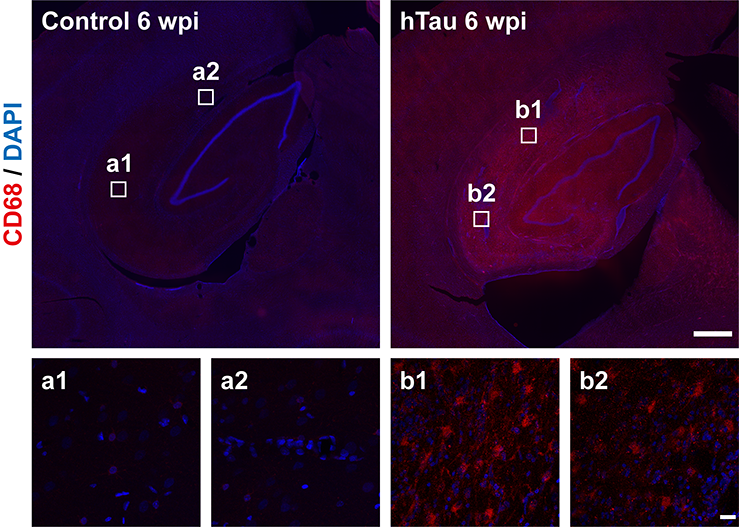

Supplement: Supplementary file 7 — Additional file 7: Additional figure 7. Representative images of CD68 immunostaining showing elevated CD68 signals across the hippocampus, further confirming robust immune activation and neuroinflammation throughout the hippocampus after 6 weeks of hTau overexpression. Insets show higher magnification of the CA3 region. Scale bar, 1 mm (top) and 20 mm (inset, bottom). [file 13195_2024_1392_MOESM7_ESM.tif]
